# A new combination strategy to enhance apoptosis in cancer cells by using nanoparticles as biocompatible drug delivery carriers

**DOI:** 10.1038/s41598-021-92447-x

**Published:** 2021-06-22

**Authors:** Ertan Kucuksayan, Fatih Bozkurt, Mustafa Tahsin Yilmaz, Aslinur Sircan-Kucuksayan, Aysegul Hanikoglu, Tomris Ozben

**Affiliations:** 1 Faculty of Medicine, Department of Medical Biochemistry, Alanya Alaaddin Keykubat University (ALKU), Antalya, 07490 Turkey; 2 Chemical and Metallurgical Engineering Faculty, Department of Food Engineering, Yildiz Technical University, Istanbul, Turkey; 3 Faculty of Engineering, Department of Industrial Engineering, King Abdulaziz University, Jeddah, 21589 Saudi Arabia; 4 Faculty of Medicine, Department of Biophysics, Alanya Alaaddin Keykubat University (ALKU), Antalya, 07490 Turkey; 5 Faculty of Medicine, Department of Medical Biochemistry, Akdeniz University, Antalya, Turkey; 6 Faculty of Engineering and Architecture, Department of Food Engineering, Mus Alparslan University, Mus, Turkey

**Keywords:** Biochemistry, Biological techniques, Biophysics, Biotechnology, Cancer, Cell biology, Chemical biology, Molecular biology, Physiology, Nanoscience and technology

## Abstract

Some experimental and clinical studies have been conducted for the usage of chemotherapeutic drugs encapsulated into nanoparticles (NPs). However, no study has been conducted so far on the co-encapsulation of doxorubicin (Dox) and epoxomicin (Epo) into NPs as biocompatible drug delivery carriers. Therefore, we investigated if co-encapsulation of doxorubicin (Dox) and/or epoxomicin (Epo) into NPs enhance their anticancer efficiency and prevent drug resistance and toxicity to normal cells. We synthesized Dox and/or Epo loaded poly (lactic-co-glycolic acid) (PLGA) NPs using a multiple emulsion solvent evaporation technique and characterized them in terms of their particle size and stability, surface, molecular, thermal, encapsulation efficiency and in vitro release properties. We studied the effects of drug encapsulated NPs on cellular accumulation, intracellular drug levels, oxidative stress status, cellular viability, drug resistance, 20S proteasome activity, cytosolic Nuclear Factor Kappa B (NF-κB-p65), and apoptosis in breast cancer and normal cells. Our results proved that the nanoparticles we synthesized were thermally stable possessing higher encapsulation efficiency and particle stability. Thermal, morphological and molecular analyses demonstrated the presence of Dox and/or Epo within NPs, indicating that they were successfully loaded. Cell line assays proved that Dox and Epo loaded NPs were less cytotoxic to single-layer normal HUVECs than free Dox and Epo, suggesting that the NPs would be biocompatible drug delivery carriers. The apoptotic index of free Dox and Epo increased 50% through their encapsulation into NPs, proving combination strategy to enhance apoptosis in breast cancer cells. Our results demonstrated that the co-encapsulation of Dox and Epo within NPs would be a promising treatment strategy to overcome multidrug resistance and toxicity to normal tissues that can be studied in further in vivo and clinical studies in breast cancer.

## Introduction

Breast cancer is the most common cancer ranking first to cause deaths from cancer in women worldwide (Global Health Estimates, WHO 2018). Chemotherapy is widely used as an important treatment modality in many cancers. Chemotherapeutic drugs are vital in the treatment and monitoring of both initial and advanced breast cancer. However, drug resistance may develop against chemotherapy in most of the cases resulting failure in treatment^1^. Therefore, increasing the effectiveness of chemotherapy is an important step to overcome drug resistance developed in cancer patients. Antineoplastic drugs possessing different mechanisms are generally used in different combinations to achieve a synergistic effect. Chemotherapeutic agents might have some limitations decreasing their effectiveness. The hydrophobicity of chemotherapeutic drugs and their low solubility, entrapment of these drugs into cell membranes is an important limitation. The other limitation might be their size preventing them to permeate and diffuse thoroughly into the cells to show their effectiveness. Another limitation is the damage they may give to healthy tissues and development of drug resistance against them^2^. In the last decades, different techniques and methodologies have been tested and applied to cope with these limitations. Application of nanotechnological techniques such as nanocarriers offers solutions to these limitations transporting anti-cancer agents within nanoparticles (NPs)^3^.

Doxorubicin (adriamycin, Dox) is an anthracyclin-type (antineoplastic) broad spectrum antibiotic used in chemotherapy to treat various cancer types including breast cancer^4–6^. The Dox-induced lipid peroxidation results in decreased membrane potential and fluidity, increased ion permeability and consequently cell death^7^. Dox has many negative side effects such as heart failure, nephrotoxicity, and extravasation injury. To overcome such side effects, the NPs loaded with Dox have been recently tested in a number of studies providing a great potential for safer drug delivery applications^8,9^.

Nuclear Factor Kappa B (NF-κB) is responsible for the transcriptional regulation of more than 150 genes involved in migration, invasion, survival, cell proliferation and apoptosis escape processes; therefore, it plays a critical role in carcinogenesis^10^. NF-κB as a transcription factor is continuously active in most aggressive tumors and capable of regulating proteins associated with the progression of epithelial cancers such as lung and breast cancers^11^. In breast cancer, NF-κB is highly activated, which leads to the development of excessive resistance to cancer treatment^12^. Due to their proteasomal degradation effects, many NF-κB inhibitors like Inhibitor Kappa B (I-κB) are used to prevent activation of NF-κB at pre-clinical level. These inhibitors can significantly reduce proliferation of breast cancer cells. However, the high complexity of the NF-κB signaling pathway and inadequate understanding of the functional mechanisms of the factors involved in this pathway pose an obstacle for the application of NF-κB inhibitors in clinical trials^13^.

The proteasome is a large multi-catalytic protease that is responsible for degradation of the majority of intracellular proteins^14^. Several key regulatory proteins involved in cell proliferation and differentiation are regulated by proteasome-mediated proteolysis, which results in the activation or inhibition of specific cell signaling pathways^15^. The proteasome is also central to the regulation of cell death and apoptosis^16^. Proteasome complex (26S) is responsible for the degradation of approximately 80–90% of intracellular proteins such as transcription factors, important proteins involved in cell apoptosis, p53, NF-κB, I-κB and oncogenes, which play a key role in cell vitality^17,18^. Therefore, proteasome inhibition may be an efficient and important treatment approach to stimulate apoptosis in breast cancer cells.

Epoxomicin (Epo) is a natural selective proteasome inhibitor, having anti-inflammatory effect. Carfilzomib is an Epo derivative and proteasome inhibitor. It was approved by U.S. Food and Drug Administration (FDA) in 2012 for the treatment of patients with recurrent resistant multiple myeloma^19,20^. Despite its use in cancer treatment, there is still not enough information in the literature regarding the role of Epo on the drug resistance developed in the treatment of breast cancer. Co-administration of Epo with potent Dox can reduce the side effects of Dox on normal tissues because of their synergistic therapeutic effect and consequently reduction of the required amount of Dox and other anticancer agents. For this reason, we established a hypothesis in our study to see if Epo may overcome the drug resistance by suppressing NF-κB activation by proteasome inhibition in breast cancer and investigated it.

The NPs have been shown to be very useful in the drug delivery inside the cells due to their high biocompatibility and ability to maintain delivery in the treatment of both advanced and early breast cancers. A drug combination therapy based on the nano-sized drug delivery system can be a promising strategy by combining drugs having two or more anti-tumor mechanisms. The Polymeric-based NPs have been gaining attention due to their biodegradability and non-cytotoxicity in the recent years. PLGA or poly(lactic-co-glycolic acid) is a biocompatible polymer that can be used in successful encapsulation of the hydrophobic drugs; facilitating an increase in the retention time of the drug in plasma^21^. In our study, we co-encapsulated Dox and Epo in PLGA based NPs fabricated using a multiple emulsion solvent evaporation technique. The synthesized NPs were characterized with respect to their particle size and stability, surface morphology, molecular, thermal, encapsulation efficiency and in vitro drug release properties. In addition, the cell line assays were performed to demonstrate the effects of the synthesized NPs on cellular accumulation, oxidative stress status, cytotoxicity, apoptosis, drug resistance and apoptotic pathways and apoptotic mechanisms in breast cancer (MCF-7) and normal human umbilical vein endothelial cells (HUVECs).

## Results

### Particle size and zeta potential values of NPs

The NPs were lyophilized using a freeze dryer for 2–3 days to maintain their stability, avoid degradation and prepare solutions at targeted concentrations. The states of NPs in the powder form and the form dissolved in phosphate-buffered saline (PBS) for use in cell culture assays are shown in Supplementary Data, respectively. The particle size of free NPs (not loaded with drug) was measured as 144.9 nm, while their sizes were observed to increase to the levels ranging from 162.1 to 179.6 nm (Table 1) when they were loaded with Dox and/or Epo, demonstrating that the drugs were successfully loaded (Supplementary Data) within the nanoparticles. NPs were synthesized in the sizes ranging between 100 and 200 nm that is a targeted range for clinical applications. The zeta potential value of free NPs was determined to be − 9.51 mV and this value was significantly increased to above − 11 mV (Table 1) when Dox was loaded into NPs, which shows that the particle stability was partly increased. The polydispersity index (PDI) value of all the nanoparticles was less than 0.01. These values are ideal for clinical applications in which NPs are not desired to be agglomerated. Additionally, after the NPs were dispersed with PBS, no agglomeration or precipitation was observed in the cell culture experiments.

**Table 1:** Average particle size and zeta potential values of the synthesized NPs.

Groups	PDI	Size (nm) ± SD	Zeta potentials (mV) ± SD
Free-NPs	0.034	144.9 ± 37.52	-9.51 ± 0.316
Epo-NPs	0.058	175 ± 51.9*	-9.72 ± 0.509
Dox-NPs	0.075	162.1 ± 55.44*	-11.3 ± 1.22*
Dox + Epo-NPs	0.047	179.6 ± 53.87*	-11.1 ± 0.115*

*Values of treatment groups are significantly different from those of free-NPs (p < 0.05).

### Surface morphology of NPs

**Figure 1:**
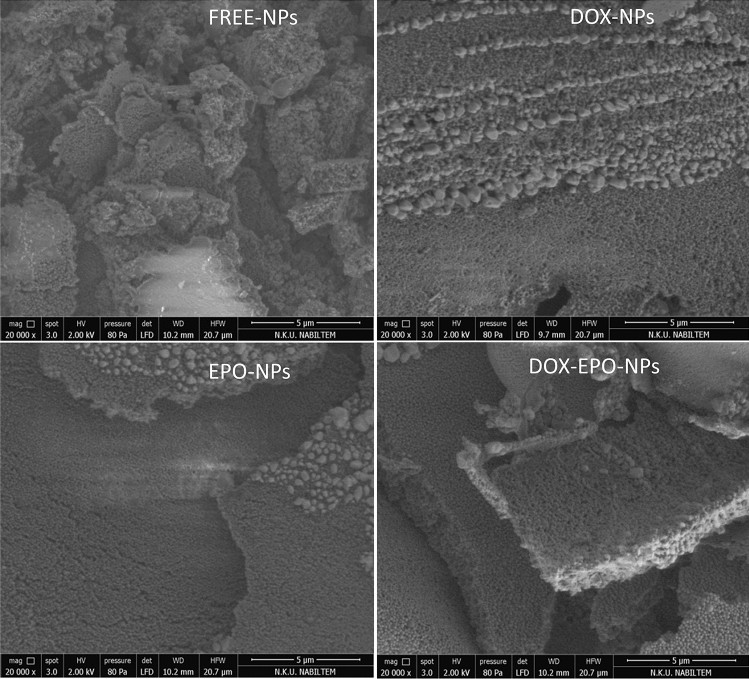
SEM images of free, Dox loaded NPs, Epo loaded NPs, and Dox-Epo loaded NPs.

The scanning electron microscope (SEM) images of free and drug-loaded NPs revealed their regular spherical shape with a narrow and unimodal size distribution and show that they were stable, homogenous, and smooth with a solid surface. The particle sizes in the images were analyzed using an Image J program and their sizes were found in the range of 164–225 nm (Table 2). SEM images showed that the sizes and shapes of NPs did not change after drug incorporation, which was in accordance with the results determined by particle size analysis (Fig. 1).

**Table 2:** Mean size values of NPs as determined by SEM image analysis.

Groups	SEM size (nm) ± SD
Free-NPs	164 ± 11.64
Epo-NPs	182 ± 16.3*
Dox-NPs	165 ± 13.1*
Dox + Epo-NPs	225 ± 15.2*^,#^

*Values of treatment groups are significantly different from those of free-NPs (p < 0.01).

^#^Value of Dox + Epo-NPs is significantly different from those of the Dox-NPs and Epo-NPs (p < 0.01).

### Molecular characterization of NPs

**Figure 2:**
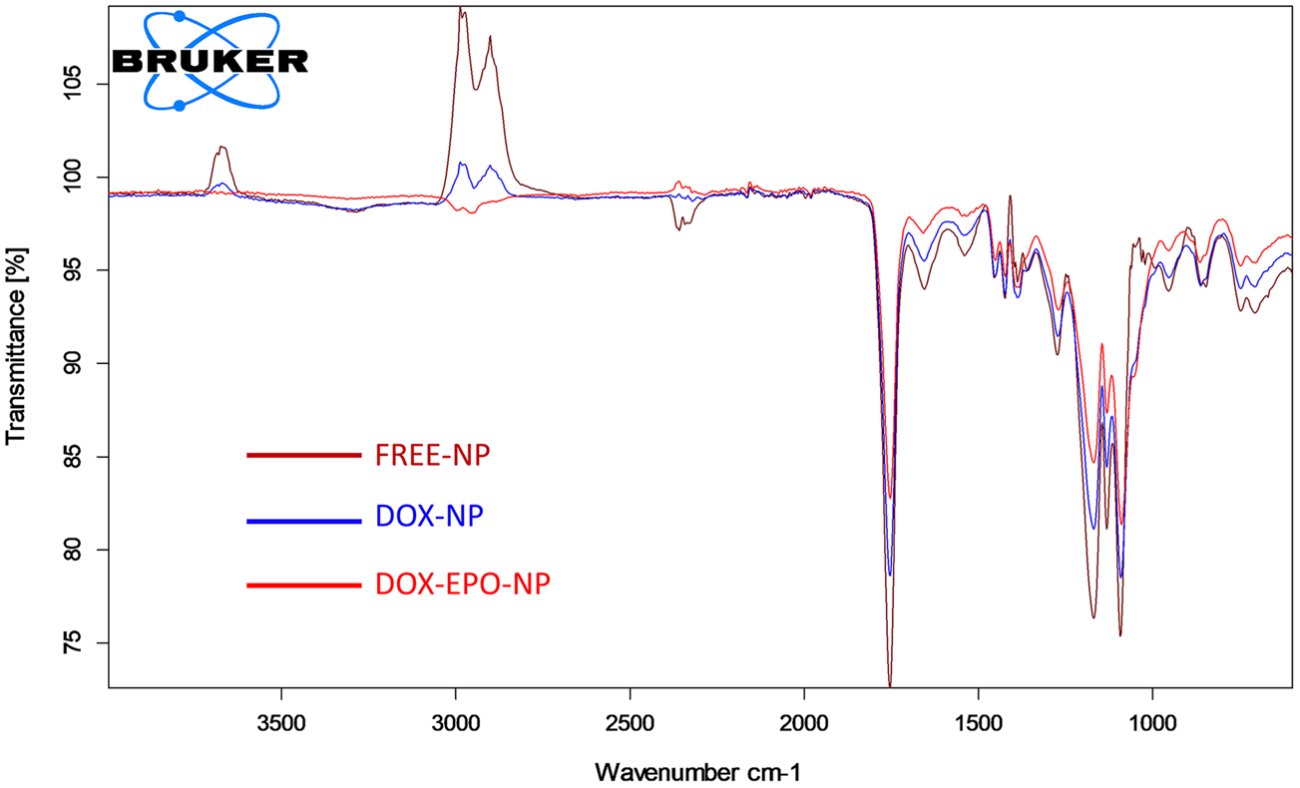
ATR-FTIR spectra of free NPs and NPs loaded with drugs.

Attenuated Total Reflection-Fourier Transform Infrared (ATR-FTIR) Spectroscopy analysis was performed to detect intermolecular chemical interactions and structural changes of NPs (Fig. 2). The NPs prepared in this study consisted of PLGA, lactide and glycolide monomers; therefore, in determination of the characteristic peaks of the copolymer, C=O, C–O, CH_3_, CH_2_ and C-H groups were taken as reference^22^. The peak observed at 1750 cm^–1^ was assigned to the carbonyl group present in both monomers of the copolymer. The intensity of this peak weakened due to the hydroxyl groups present in both Dox and Epo loaded within PLGA. There were also bands observed at 1300 and 1150 cm^–1^ which were caused by symmetrical and asymmetrical C–C (=O) –O vibrations. These peaks are used for characterization of esters. Thus, this strain weakened in the NPs loaded with both drugs. The absorption band between 3600 cm^–1^ and 3000 cm^–1^ which was assigned to the hydroxyl group appeared in the spectrum of NPs loaded with Dox (Dox-NPs), while this slight peak disappeared in the spectrum of NPs loaded with Dox + Epo (Dox + Epo-NPs). In the spectrum of free-NPs, the aliphatic C-H, O–H and N–H stretching vibration peaks were observed between 3000 cm^–1^ and 2850 cm^–1^. However, loading of the Dox and Epo within the NPs resulted in the formation of O–H and N–H stretching peaks. Furthermore, this peak was slightly stronger in the spectrum of Dox + Epo-NPs group than in the spectra of free-NPs and Dox-NPs, which showed that Dox + Epo was also successfully loaded into NPs. Accordingly, N–H bonds were only observed in the spectra of NPs separately loaded with Dox and Epo. The ATR-FTIR spectroscopy analysis revealed that Dox and/or Epo were successfully encapsulated into the PLGA based NPs.

### Thermal characterization of NPs

**Figure 3:**
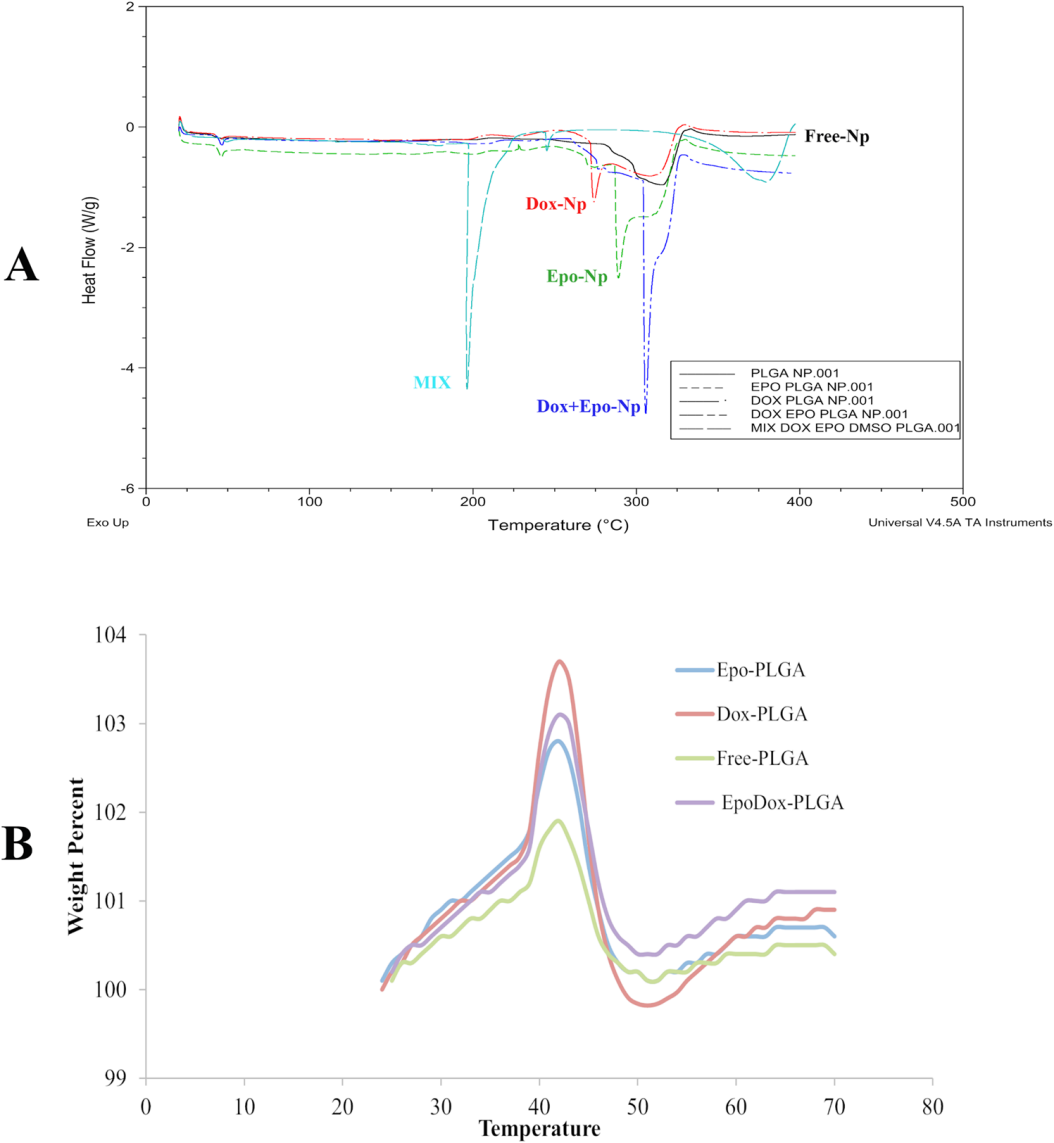
**(A)** Characterization of NPs by DSC. (**B)** Characterization of NPs by TGA.

T_o_, T_p_, and T_e_ peak temperatures were determined by performing differential scanning calorimeter (DSC) analysis. The peaks expressing endothermic heat flow were observed in thermograms of all samples. There were differences between the peak values (Table 3). The differences between the thermograms of the drug loaded NPs were in terms of endothermic peaks observed in the glassy passivity region. The endothermic peak of Free-NPs was observed at lower level than those of NPs loaded with the drugs (Fig. 3A). These values showed that the drug loaded NPs were more resistant to temperature than Free-NPs, which was attributed to the toughest chemical bonding in Dox + Epo-NPs.

**Table 3:** DSC values for all nanoparticles. T_o_ (temperature at which peak starts), T_p_ (peak temperature) and T_e_ (temperature at which peak ends).

Groups	T_o_ °C	T_p_ °C	T_e_ °C
Free-NPs	40.56	200.57	278.47
Epo-NPs	43.16*	200.39	271.55*
Dox-NPs	42.29*	210.73*^,#^	273.76*
Dox + Epo-NPs	43.67*	205.76*^,#^	277.27

Mean temperature of NPs by DSC.

*Values significantly different from Free-NPs p < 0.001.

^#^Values significantly different from Free-NPs and Epo-NPs p < 0.001.

Thermal gravimetric analysis (TGA) was conducted to determine decomposition temperature (Td) values from 0 to 750 ºC. No remarkable difference between the Td values of the samples was recorded, which might be since the drugs were loaded in low amounts. The difference in the mass change at the initial decomposition temperatures was determined, which shows the glassy permeability region of the NPs (Fig. 3B). It was found that the percentage masses of the NPs were different at the initial decomposition temperature of 42 °C due to the loading of Dox and / or Epo. The glassy pass-through drugs varied depending on the region of the endothermic peaks in the thermograms of drug loaded NPs. As a result, thermal characterization analysis confirmed that Dox and / or Epo drugs were loaded into the NPs.

### Encapsulation efficiency and in vitro release of drugs

We calculated separately the efficiency of the encapsulation of Dox-NPs, Epo-NPs, and Dox + Epo-NPs. The efficiency of the encapsulation of Dox was 79.2 ± 0.69% at Dox-NPs (w/w). The efficiency of the encapsulation of Epo was 83.56 ± 0.78% at Epo-NPs (w/w). In addition, we calculated in Dox + Epo-NPs, the NPs loaded with Dox and Epo, 77.8 ± 0.88% and 82.4 ± 0.74%, respectively. Since both drugs are hydrophobic, they are highly encapsulated into PLGA, an amphipathic molecule.

**Figure 4:**
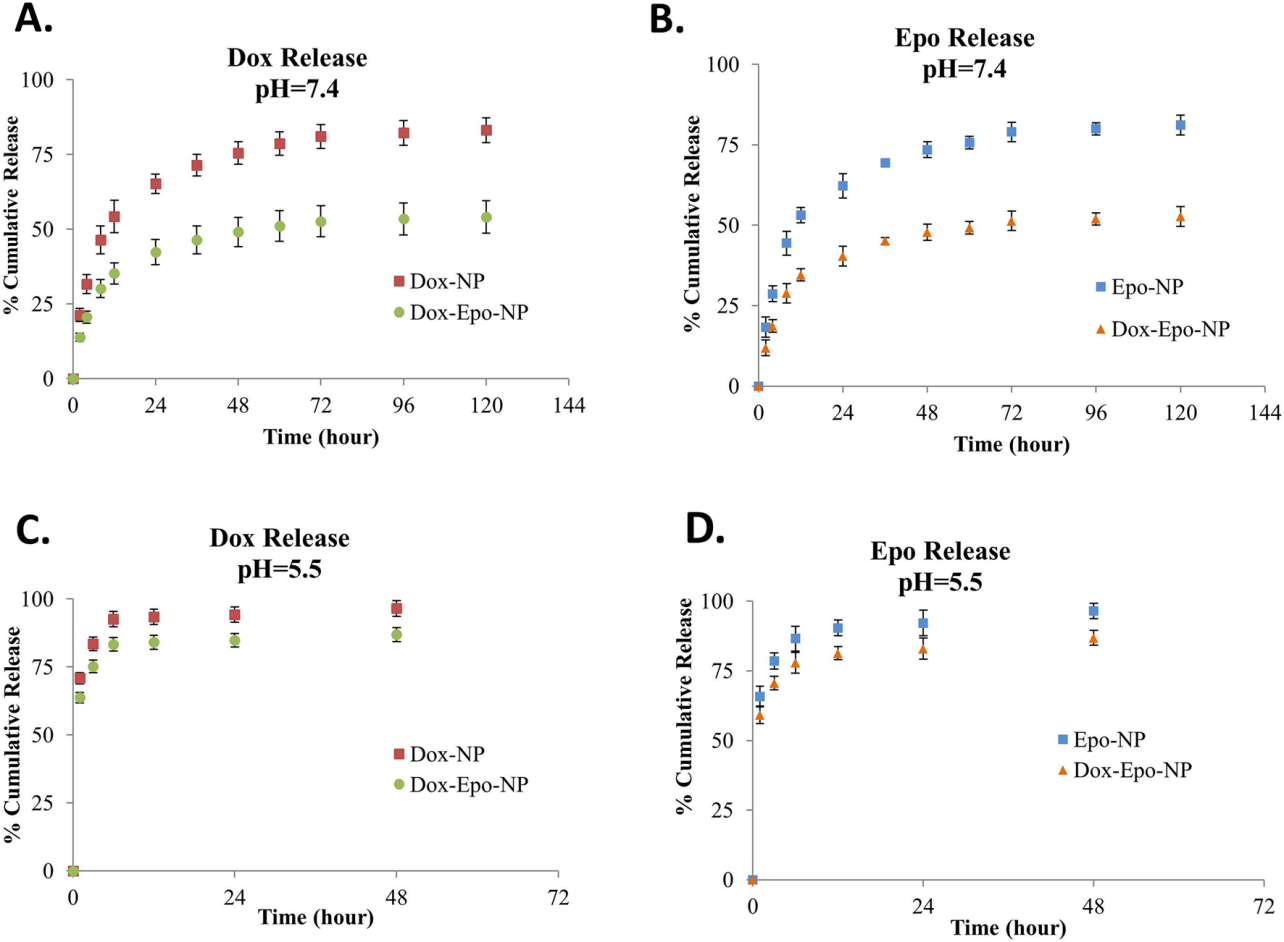
**(A)** Cumulative release of Dox versus time from two different Dox + Epo-NPs (circle) and Dox-NPs (square) at pH 7.4; (**B)** Cumulative release of Epo versus time from two different Dox + Epo-NPs (triangle) and Epo-NPs (square) at pH 7.4; (**C)** Cumulative release of Dox versus time from two different Dox + Epo-NPs (circle) and Dox-NPs (square) at pH 5.5; (**D)** Cumulative release of Epo versus time from two different Dox + Epo-NPs (triangle) and Epo-NPs (square) at pH 5.5.

Amounts of drugs retained and released in NPs at pH 7.4 and pH 5.5 were determined in the samples taken at time intervals. These pHs for the release of the NPs are chosen because pH  7.4 and pH 5.5, represent physiological pH and lysosomal pH inside cells, respectively. The released Dox and Epo levels were quantified using high-performance liquid chromatography (HPLC) and liquid chromatography / mass spectrometry-quadrupole time-of-flight) (LC–MS-QTOF), respectively. In Fig. 4A, we found that Dox-NPs and Dox + Epo-NPs groups with Dox loaded had 50% rapid release of Dox in 24 h and slow release of Dox after 24 h at pH  7.4. Similarly, we found that Epo-NPs and Dox + Epo-NPs groups with Epo loaded had 50% rapid release of Epo in 24 h and slow release of Epo after 24 h at pH  7.4. (Fig. 4B). In contrast, Dox-loaded Dox-NPs and Dox + Epo-NPs groups had 50% of the Dox release within 1 h, and the whole was released at the sixth hour at pH  5.5 (Fig. 4C). Similarly, Epo loaded Epo-NPs and Dox + Epo-NPs groups had 75% of the Epo release within 2 h, and the whole was released at the twelfth hour at pH  5.5 (Fig. 4D). The results exhibited pH dependent in vitro drug release. It could be due to degradation of PLGA at low pH due to hydrolysis of the ester bonds in the polymer chains, which could facilitate the release of Epo and Dox. In addition, Dox solubility in water might increase due to the protonation of Dox as the pH decreased and increased hydrophilicity could lead to Dox to be expelled from the hydrophobic PLGA core. As a result, we evaluated that the encapsulated NPs release drugs at slow and low concentrations as desired under normal physiological conditions, while releasing at high and high concentrations at lysosomal pH.

### Cell viability studies

The investigation of the toxicity profile of the drug delivery system is of prime importance in the translation for biomedical applications. The viability and biocompatibility were evaluated in the HUVECs and the MCF-7 cells at various concentrations of the free drugs and all NPs for 24 h. IC_50_ values (half-value of the concentration that could lead to a complete inhibition) was calculated using the dose-inhibition multiple slope equation with GraphPad Prism 5 program. We have given IC_50_ values of all groups in the HUVECs and the MCF-7 cells for 24 h in Table 4. In addition, we studied the dose–response curves of all groups and found the IC_30_, IC_50_, and IC_70_ doses. The interaction between free Dox and free Epo was studied using the median-drug effect analysis. At a fixed concentration ratio between the drugs based on the IC_50_ concentration the average combination index (CI) for MCF-7 cell lines showed antagonism (CI: 1.4–2.0). However, we determined synergism between Free-NPs and Dox + Epo-NPs, which are the nanoparticle encapsulated versions of both drugs for MCF-7 cell lines (CI < 1.0).

**Table 4:** IC_50_ values of all HUVECs and MCF-7 cells groups incubated for 24 h.

Groups (µg/mL)	Dox	Epo	Dox + Epo	DOXIL	Free-NPs	Epo-NPs	Dox-NPs	Dox + Epo-NPs
HUVEC (H-IC_50_)	3.924	0.02693	2 + 0.020	114.3	4279	2547	1804	1695
MCF-7 (M-IC_50_)	1.328	0.0222	1 + 0.015	144.8	2015	1558	1423	233.3

Our goal was to induce apoptosis in the breast cancer cells, while not damaging normal cells. Hence, we used the IC_50_ (M-IC_50_) values calculated for the MCF-7 cells in all the cell culture experiments since our aim was not to harm normal cells but to induce apoptosis in breast cancer cells. For this reason, M-IC_50_ doses administered in all groups were toxic to the MCF-7 cells, but not toxic to the normal HUVECs.

### Cellular uptake and ROS generation

**Figure 5:**
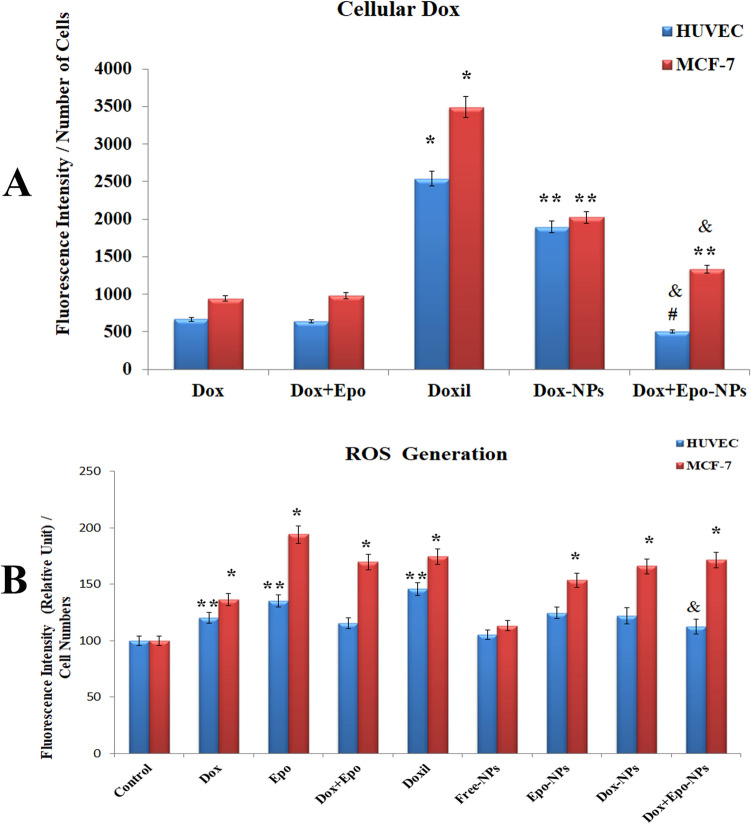
**(A)** Amounts of Dox in the nine HUVECs and MCF-7 cell groups incubated for 6 h (n = 3 in each experiment). Values are mean ± SD. *Values significantly different from all groups in the HUVECs and MCF-7 cells p < 0.001. **Values significantly different from all groups in the HUVECs and MCF-7 cells p < 0.01. ^&^Values significantly different from all groups in the HUVECs and MCF-7 cells p < 0.01. ^#^Values significantly different from all groups in the HUVECs p < 0.001. (**B)** Measurement of the ROS Generation in the nine HUVECs and MCF-7 cell groups incubated for 18 h (n = 3 in each experiment). Values are mean ± SD. *Values significantly different from control, free Epo, free Dox + Epo and all NPs groups in HUVECs p < 0.001. **Values significantly different from control, free-NPs, and Dox + Epo-NPs groups in HUVECs p < 0.01. ^&^Values significantly different from free Dox, free Epo, DOXIL in the HUVECs and MCF-7 cells p < 0.01.

Cellular internalization of the NPs was measured by taking advantage of the fluorescence feature of Dox taken into the cell. We demonstrated that the NPs containing Dox entered into the MCF-7 cells more than HUVECs in all groups, and commercial liposomal DOXIL contains more Dox than the synthesized polymeric NPs (p < 0.01). We found that Dox uptake of HUVECs was 2.5 times less than MCF-7 cells in Dox + Epo-NPs group (p < 0.001) (Fig. 5A). In addition, we showed that the Dox + Epo-NPs group had a statistically significant lower intake of Dox into the cell than the free Dox and free Dox + Epo groups in the HUVECs (p < 0.001). So, Dox entering the cell with Dox + Epo-NPs is less toxic to normal cells.

After incubation with M-IC_50_ doses, the amount of reactive oxygen species (ROS) was measured in both cell lines. It was found that Free-NPs group did not cause an increase in the ROS amount in both cell lines. However, ROS generation aggravated in all drug-containing groups in both cell lines (p < 0.001). Dox and Epo increased ROS generation indirectly. We found that ROS generation in Epo-NPs, Dox-NPs and Dox + Epo-NPs groups were higher in MCF-7 cells than HUVECs (p < 0.001) (Fig. 5B). Our results indicate that that the redox balance of the cells was disrupted, and the apoptotic pathways were activated with the combined application of the drugs in the cells.

### Inhibition of proteasome and NF-κB pathways

**Figure 6:**
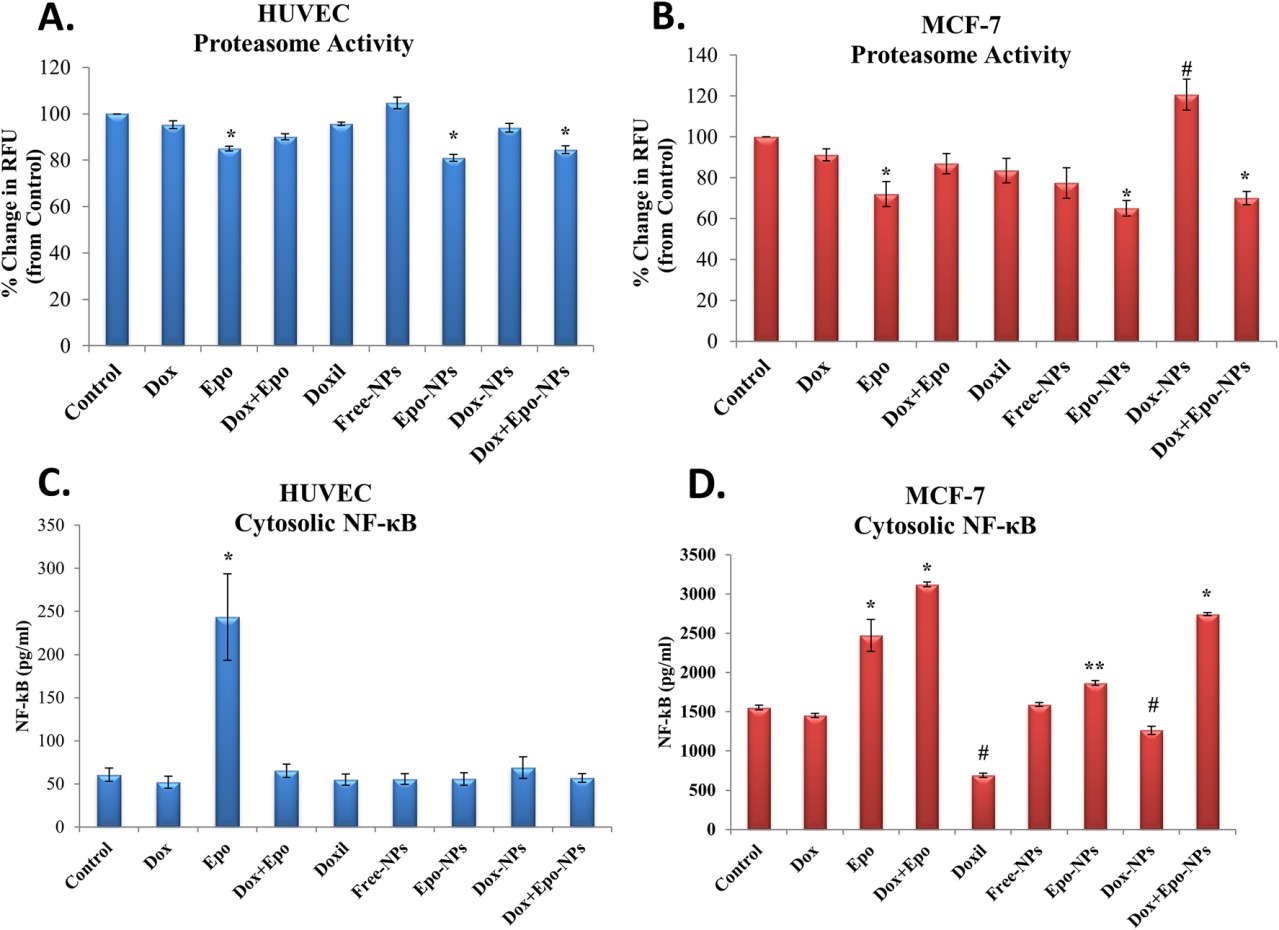
**(A)** Proteasome Activity in the nine HUVECs groups incubated for 24 h (n = 3 in each experiment). Values are mean ± SD. (**B)** Proteasome Activity in the nine MCF-7 cell groups incubated for 24 h (n = 3 in each experiment). Values are mean ± SD. *Values significantly different from the control, free Dox, free Dox + Epo, DOXIL, Free-NPs, and Dox-NPs in the HUVECs and MCF-7 cells p < 0.01. ^#^Values significantly different from control, free Dox, in the MCF-7 cells p < 0.001. (**C)** Cytosolic NF-κB levels in the nine HUVECs groups incubated for 24 h (n = 3 in each experiment). Values are mean ± SD. *Values significantly different from all groups in the HUVECs p < 0.001. (**D)** Cytosolic NF-κB levels in the nine MCF-7 cell groups incubated for 24 h (n = 3 in each experiment). Values are mean ± SD. *Values significantly different from control, free Dox, DOXIL, Free-NPs, Epo-NPs, and Dox-NPs groups in the MCF-7 cells p < 0.001. **Values significantly different from control, free Dox, DOXIL, and Free-NPs groups in the MCF-7 cells p < 0.01. ^#^Values significantly different from control, free Dox, and DOXIL groups in the MCF-7 cells p < 0.01.

Epo is a proteasome inhibitor having important natural, anti-cancer, and anti-inflammatory properties^23,24^. After incubation of the HUVECs and MCF-7 cells with the M-IC_50_ doses proteasome activity was measured by fluorometric peptidase assay. We investigated the extent and effect of cell exposure of IC_50_ concentrations for 24 h to all groups on proteasome activities. The presence of proteasome inhibition in the Epo-NPs and Dox + Epo-NPs groups showed that Epo was encapsulated into the NPs (Fig. 6A). While inhibition of proteasome was 30–35% for free Epo, Epo-NPs, and Dox + Epo NPs groups in the MCF-7 cells, it was about 10% for the same groups in the HUVECs (Fig. 6A,B). Statistically, inhibition of proteasome in Epo-NPs and Dox + Epo-NPs groups was found to be higher in the MCF-7 cells than the HUVECs (p < 0.001). These results are also compatible with cytotoxicity data. Proteasome inhibition was not observed in groups without Epo. Interestingly in the MCF-7 cells, we found that proteasomal activity increased in the Dox-NPs group. This result supported the theory that cancer cells need new proteins to activate drug resistance mechanisms (Fig. 6B). Therefore, targeting proteasomes in combined chemotherapy applications is an important novel strategy in the breast cancer.

Epo entering the cells via NPs, suppressed the nuclear translocation of NF-κB and kept it in the cytosol by proteasomal inhibition of I-κB. We observed this more clearly, especially in the MCF-7 cell groups containing Epo (Fig. 6B,D). NF-κB suppression was highly significant in the free Epo group, but not in other groups in the HUVECs (Fig. 6A,C). This showed that the use of free Epo for treatment can be harmful to normal cells. Fortunately, encapsulation of Epo into the NPs eliminated this damage. On the other hand, NF-κB expression is known to be very high in the MCF-7 cells. The high amount of NF-κB in both Epo-NPs and Dox + Epo-NPs groups showed nanoparticles entered into the cells to prevent the degradation of I-κB, and the NF-κB was remained in the cytosol by proteasomal inhibition (Fig. 6D).

### Flow cytometric analysis

**Figure 7:**
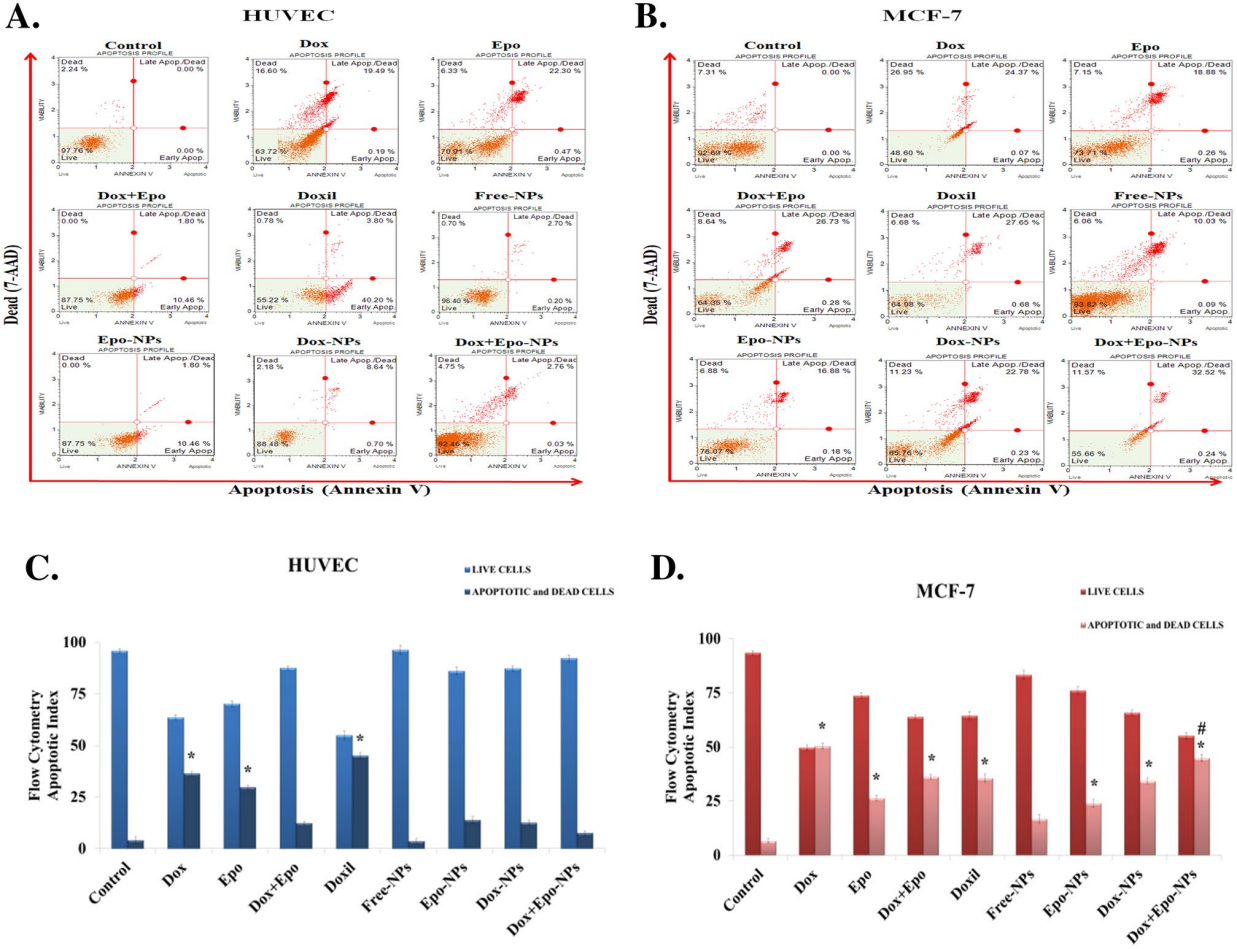
**(A)** Percentage of survival and apoptotic cells using FC analysis in the nine HUVECs groups incubated for 6 h. Values are mean ± SD, n = 3 measurements of 25.000 cells (n = 3 in each experiment). (**B)** Percentage of survival and apoptotic cells using FC analysis in the nine MCF-7 cell groups incubated for 6 h. Values are mean ± SD, n = 3 measurements of 25.000 cells (n = 3 in each experiment). (**C)** Percentage of apoptotic index in the nine HUVECs groups incubated for 6 h (n = 3 in each experiment). Values are mean ± SD. *Values significantly different from the control, free Dox + Epo, Free-NPs, Epo-NPs, Dox-NPs, and Dox + Epo-NPs groups in the HUVECs p < 0.001. (**D)** Percentage of apoptotic index in the nine MCF-7 cell groups incubated for 6 h (n = 3 in each experiment). Values are mean ± SD. *Values significantly different from the control and Free-NPs groups in the HUVECs p < 0.001. ^#^Values significantly different from control, free Dox, free Epo, free Dox + Epo, Epo-NPs, and Dox-NPs groups in the MCF-7 cells p < 0.001.

HUVECs and the MCF-7 cells were analyzed the apoptotic cells by Annexin V-PE and dead cells staining by 7-amino-actinomycin D (7-AAD). We observed that both HUVECs and MCF-7 cells were killed with free Dox and/or Epo induced apoptosis. We found that apoptosis was statistically reduced in the groups which was encapsulated with Dox and/or Epo to the NPs in the HUVECs (Fig. 7A,C). Thus, encapsulation of drugs into nanoparticles eliminated drug toxicity and prevented normal cells from apoptosis. Apoptotic index increased in Dox + Epo-NPs group compared to the free Dox and Epo combined group in the MCF-7 cells (Fig. 7B,D). While the apoptotic index was around 8% in HUVECs, this rate increased to 45% in MCF-7 cells. Flow cytometry data showed that the MCF-7 cells could not escape from apoptosis and they died in the Dox + Epo-NPs group, while HUVECs remained alive in the same group.

### TUNEL analysis

**Figure 8:**
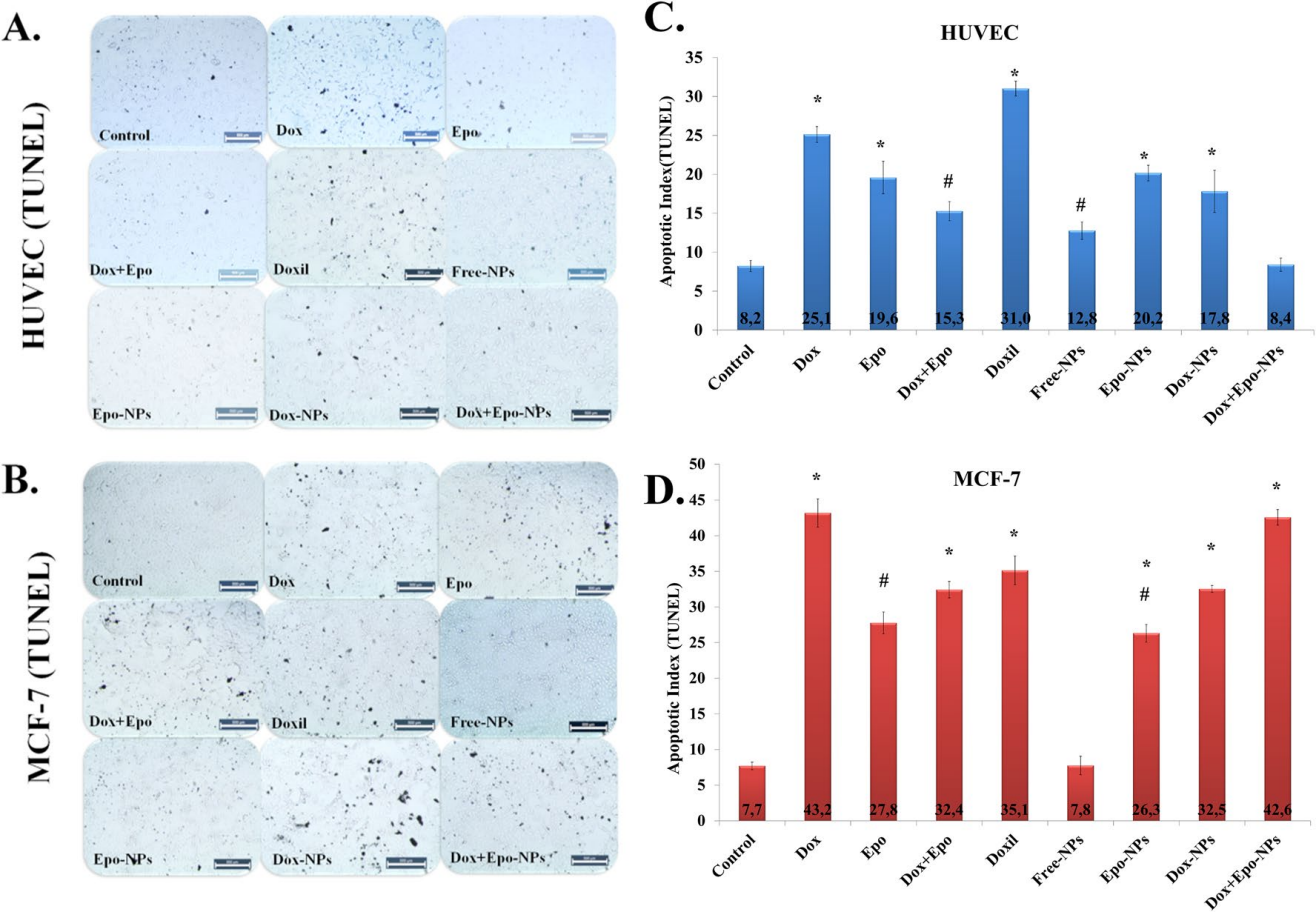
**(A,B)** Representative images showing apoptotic HUVECs and MCF-7 cells in the nine groups visualized by TUNEL-staining (dark brown) after incubation for 11 h. Scale bar represents 500 µm; magnification ×4. (**C)** Percentage of apoptotic index in the nine HUVECs groups incubated for 11 h. Values are mean ± SD, n = 3 measurements of 10.000 cells (n = 3 in each experiment). *Values significantly different from the control, free Dox + Epo, Free-NPs, and Dox + Epo-NPs groups in the HUVECs p < 0.001. ^#^Values significantly different from control, free Dox, free Epo, Epo-NPs, and Dox-NPs groups in the HUVECs p < 0.001. (**D)** Percentage of apoptotic index in the nine MCF-7 cell groups incubated for 6 h (n = 3 in each experiment). Values are mean ± SD. *Values significantly different from the control and Free-NPs groups in the MCF-7 cells p < 0.001. #Values significantly different from control, free Dox, free Dox + Epo, DOXIL, Dox-NPs, and Dox-Epo-NPs groups in the MCF-7 cells p < 0.001.

We kept the incubation time longer in the Annexin V method to detect apoptosis in “terminal deoxynucleotidyl transferase (TdT)-mediated dUTP-biotin nick-end-labeling” (TUNEL) analysis. Early apoptosis is detected in the Annexin V method, while DNA fragmentation which is the last stage of apoptosis is seen in the TUNEL method. We also morphologically showed the effects of incubation with M-IC_50_ doses on the HUVECs. TUNEL staining results and flow cytometry data were compatible with each other. While free drugs had cytotoxic effects both on cancer and normal cells. The Dox + Epo-NPs group had sixfold cytotoxic effects on breast cancer cells compared to the controls. It did not have cytotoxic effects on the normal HUVECs (Fig. 8).

## Discussion

Nanotechnology based drug carriers of single or combined drugs, are designed and tested as anticancer agents to achieve high survival time for the various cancers^25–27^. The synthesis and preclinical studies of the NPs with different features are continuing intensively in order to shorten the treatment processes and increase the efficiency of the treatments and patients’ well-being^28,29^. A number of nanoparticle-based formulations have been developed, such as inorganic NPs, polymeric drug micelles, liposomes, dendrimers, carbon nanotubes, and nanorods^30,31^. However, while developing a treatment strategy using nanomedicine, it should be aimed to minimize the harm to the patient. Specifically the FDA approval of DOXIL highlights the advantages of nanodrugs by making it possible to diminish the serious side effects, mainly the cardiac toxicity, and elevate the general patient health status^32^. DOXIL has been unsuccessful to demonstrate significant advance in the rate of progress in survival rates in clinical settings. In our study, we observed similar toxicity of Dox in the normal cells. One of the approaches for reduction of the toxic effects of chemotherapeutic drugs on normal cells and enhancement of their antitumor efficiency is supposed to use of the biopolymer delivery systems. Polymeric NPs carriers have several advantages compared to other nano-sized drug carriers including good tumor accumulation, easy preparation, biocompability, biodegradability and easy modification by targeting ligands^31^.

Tumor microenvironment due to vascular angiogenesis usually creates a space ranging between 100 and 2000 nm. Therefore, in study, we synthesized all NPs in 100–200 nm size in order to be able to passive targeting, which can pass through the endothelial space in the cancer region and also prevent to be filtered from the kidneys. There is a selective uptake of nanoparticles in MCF-7 cells rather than normal HUVECs due to the tendency of cancer cells to accumulate macromolecules besides inhibition of drug efflux^33^. Furthermore, it has also been observed that NPs smaller than 200 nm are internalized by clathrin-mediated endocytosis. In another study investigating the cell internalization of PLGA nanoparticles in epithelial cells, it was found that the expression of clathrin and caveolin proteins did not increase, but still nanoparticles entered into the cells^34^. We confirmed the NPs sizes ranging from 144.9 ± 37.52 to 179.6 ± 53.87 nm, and PDI value is in the range of 0.034–0.075 in the entire particle formulation group by zetasizer (Supplementary Data and Table 1). In addition, zeta potential values of the NPs were found to be between 9.51 and 11.3 mV. This provides that the NPs developed cannot agglomerate and are ideal for clinical applications^35^. We calculated the images of the NPs with various particle sizes ranging from 164 ± 11.64 to 225 ± 15.2 nm by SEM analysis (Fig. 1 and Table 2). So, we precisely controlled the shapes and sizes of the NPs. In recent studies, the NPs sizes prepared were in the range of 100–600 nm^36–38^.

As a result of ATR-FTIR Spectroscopic analysis of the NPs, it was shown that Dox and/or Epo entered into the PLGA-NPs. When the drugs interacted with PLGA, PLGA-specific peaks disappeared in the spectrum. Peak changes were not sharp in drug groups due to the low concentrations of drugs (Fig. 2). However, since the effective cytotoxic doses of the drugs were in the range of micrograms and/or nanograms, we were able to detect cytotoxicity in breast cancer cells. As a result of the characterization of the NPs with DSC, we found that Dox and/or Epo drugs were dispersed into the NPs. The endothermic peaks in the vitreous region of drugs were different from the loaded drug (Fig. 3A). When the Td values of the NPs were measured as a result of TGA, we observed differences in percent mass change in the Dox and/or Epo groups at the initial degradation temperature (Fig. 3B). We investigated the degradation of the NPs at different pH values to acquire a first response on the release of the drugs from the NPs. The results exhibited pH dependent in vitro drug release. According to the characterization experiments, since the drugs are hydrophobic, they are highly encapsulated into PLGA, which is an amphipathic molecule. Epo contains hydroxyl, ketone, epoxy and amino groups. So, the passage of Epo through the cell membrane within the nanoparticle is facilitated by the polarity of the cell membranes. In addition, after Dox + Epo-NPs enters into the cells and endosomal degradation begins, these chemical groups allow the nanoparticle to open easily with the decrease of pH and the effect of hydrogen bonds. We found that NPs had very low drug release at normal physiological pH 7.4 levels, but drug release increased in a short period of time at pH 5.5 levels, which is the lysosomal pH. This could be due to degradation of PLGA at low pH due to hydrolysis of the ester bonds in the polymer chains, which could facilitate the release of Epo and Dox^39^. In addition, Dox solubility in water might increase due to the protonation of Dox as the decreased pH and increased hydrophilicity could lead to Dox to be expelled from the hydrophobic PLGA core^40^. Another reason might be the change of the intrinsic viscosity of bovine serum albumin (BSA) depending on pH. Intrinsic viscosity ([*η*]) is an expression of the interaction between a biopolymer and a solvent, reflecting the solvent's ability to swell the macromolecule. Curvale et al. found that BSA possessed a very low [*η*] value at pH 7.4 which explains the assigned globular shape and [*η*] value was triplicated at pH 2.7^41^. Swelling of BSA might cause degradation of nanoparticle integrity and contribute to the release of more Epo and Dox from the nanoparticle at low pH. Consequently, the release rate of encapsulated NPs is high at lysosomal pH, while slow as desired under normal physiological conditions. Lysosomal pathway is activated by the penetration of NPs into cells. Thus, it shows that NPs release drugs into the cells when they enter and degraded (Fig. 4).

Due to the molecular complexity of cancer, our approach can synergistically increase the effects of various agents having different mechanisms of action, overcome drug resistance and provide a better successful treatment. The Dox and Epo are poorly water-soluble drugs and can be trapped in the cell membranes. Nano-sized formulation with proven longevity in the systemic circulation can unlock the mystery of NF-κB pathway and proteasome inhibition capability of Epo. In this study, PLGA based polymeric NPs co-loaded with Epo and Dox were evaluated and found to decline toxicity of free drugs in normal cells. The hydrophobic nature of Epo and Dox allowed them to be incorporated into the PLGA-NPs.

Physiologically regular proteasomal activity affects the intracellular proteome composition extensively and regulates cell proliferation, differentiation, survival and apoptosis^42,43^. Small et al. performed that the combination of a proteasome inhibitor bortezomib and Dox has been shown to reduce MKP-1 protein levels, to increase JNK activation causing cells to undergo apoptosis in breast cancer cells^44^. Similarly, Gavilan et al. found that the GSK-3β signaling pathway-induced autophagy was elevated through proteasome inhibition with MG132, which is a 26S proteasome inhibitor like Epo, in breast cancer cells^45^. MG132 has been shown to induce apoptosis effectively in various tumor cells, including osteosarcoma, small cell lung cancer, and lymphoma cells^46–48^. However, these drugs were not encapsulated into NPs and were given freely in these studies. In our study, we demonstrated that Epo stimulates apoptosis via proteasome inhibition in the breast cancer cells based on flow cytometry and TUNEL results. Free Dox and free Epo treatment caused both normal and cancer cells to undergo apoptosis. Conversely, while the Dox + Epo-NPs group induced apoptosis in half of the breast cancer cells, there was no significant apoptosis in normal cells. So, we propose that Dox + Epo-NPs treatment might be used for further experimental animal and clinical studies.

NF-κB activation has been shown to be associated with increased aggressive properties of cancer cells^49^. Kucuksayan et al. demonstrated that activation of NF-κB pathway is crucial for the survival of breast and other cancers^50^. Normally, this protein is found in the cytosol, when it passes into the nucleus, it activates regulation of more than 150 genes involved in apoptosis escape processes in cancer and causes drug resistance in cancer cells. Therefore, an increase in the amount of NF-κB in the cytosol indicates that the cells cannot escape from apoptosis and increase drug sensitive in cancer cells. As a result, drug resistance can be overcome by inhibition of proteasomal degradation of I-κB with Epo. Proteasome inhibitors have been reported to induce apoptosis in many tumor cells via ROS^46,51,52^. In our study, Dox and Epo activated ROS generation were showed by the 2ʹ,7ʹ-Dichlorofluorescin Diacetate (DCFH-DA) method. While ROS levels increased in all groups of free drugs in the normal and cancer cells, ROS decreased in the normal cell groups, and increased in breast cancer cells treated with nanoparticles. This might be interpreted that the degradation of many proteins is suppressed, the redox balance is impaired, and ROS increases through proteasome inhibition in the MCF-7 cells. According to both flow cytometry and TUNEL results in the Dox + Epo-NPs groups, apoptosis was 8% in the HUVECs, but it increased to 45% in the MCF-7 cells. So, Dox + Epo-NPs treatment is more efficient to induce apoptosis and might be used for experimental animal and clinical studies. We found that Dox + Epo-NPs were less cytotoxic in the normal HUVEC cells, while Dox + Epo-NPs were cytotoxic in the breast cancer cells. Free-NPs were found to be non-toxic even at high doses in normal cells. We examined the NPs-induced oxidative cell death by measuring proteasome inhibition, TUNEL staining and ROS levels in the HUVECs and MCF-7 cells.

**Figure 9:**
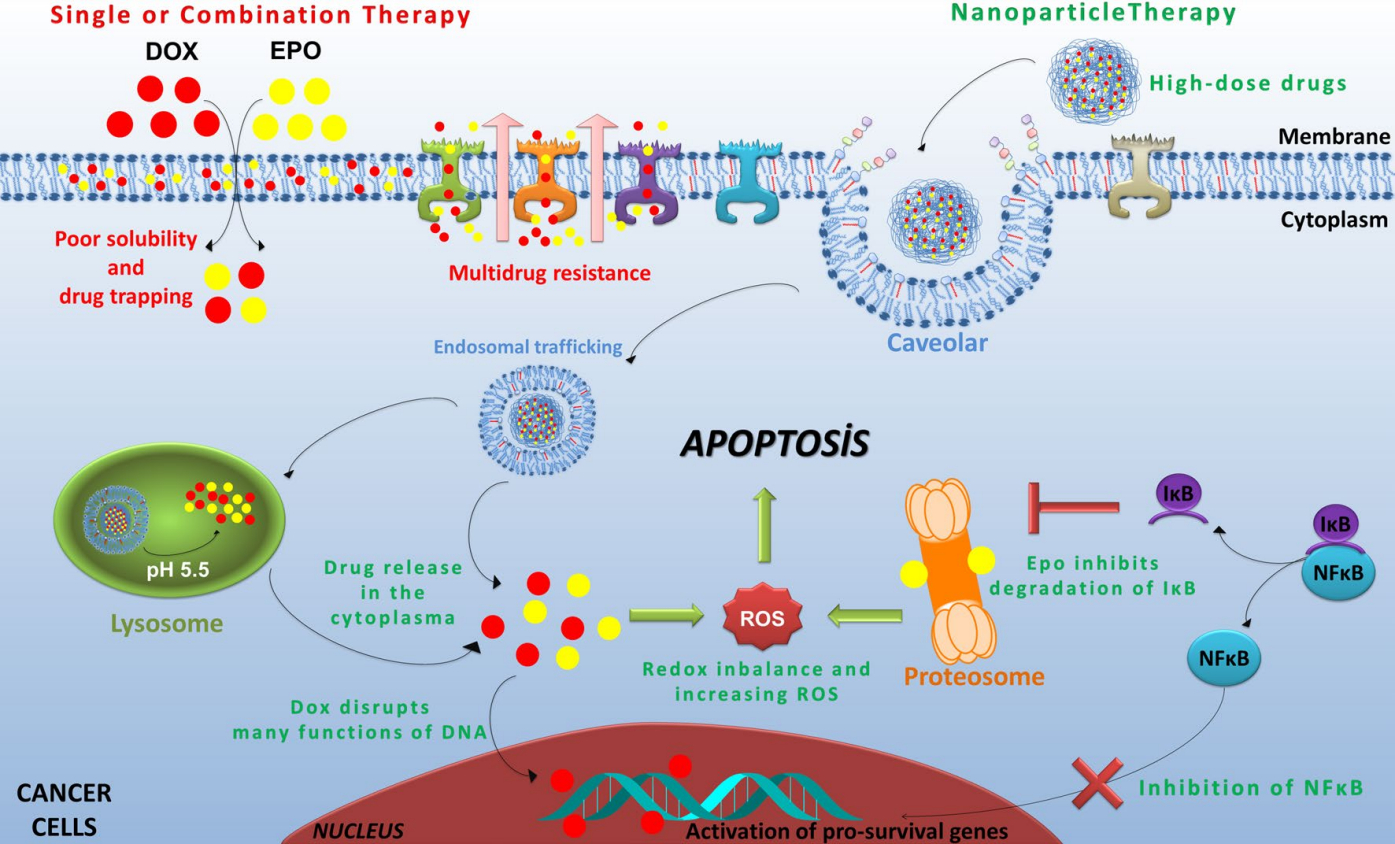
The advantages of nanoparticle therapy to single or combined application in free state are summarized in figure. Dox + Epo-NPs overcame drug resistance by suppressing NF-κB activation, proteasome inhibition, inducing apoptosis in breast cancer cells, and without exerting these effects in normal cells.

In recent years, researchers are trying to overcome multidrug resistance (MDR) such as P-glycoprotein (P-gp) dependent one with the application of different drug strategies. Zhang et al. reported that Celecoxib (CXB, a selective COX-2 inhibitor), dramatically enhances the cytotoxicity of the Dox in breast cancer cells overexpressing P-gp^53^. So, the combination of the Dox and CXB exerted synergistic effects against breast cancer by overcoming drug resistance. MG132 is an inhibitor of NF-κB and has been shown to decrease anti-apoptotic Bcl-2 and MDR1 expressions due to suppressing NF-κB activation^54–56^. In another study, Zhang et al. found that MG132 reduced drug resistance caused by vincristine, a chemotherapeutic drug, inducing apoptosis in gastric cancer cells^57^. However, many commonly used chemotherapeutic agents such as Dox causes DNA damage-induced NF-κB activation that leads to development of MDR phenotype. Wang et al. demonstrated that the P-gp levels were diminished by suppression of NF-κB through Bortezomib in leukemia cells resistant to Daunorubicin, a chemotherapeutic drug having similar effects like Dox^58^. In another study, Fujita et al. found that Bortezomib and MG132 were shown to reduce P-gp levels in breast cancer cells that are resistant to Dox and Paclitaxel^55^. On the other hand, Epo as a proteasome inhibitor like MG132 and Bortezomib, has been proposed as a promising agent in cancer treatment^59^. In this study, we found that Dox + Epo-NPs overcame drug resistance by suppressing NF-κB activation, proteasome inhibition, inducing apoptosis in breast cancer cells, and without exerting these effects in normal cells (Fig. 9).

Understanding mechanisms involved in the activation of NF-κB pathway in breast cancer is essential to target and overcome drug resistance brought by the hyperactivation of this pathway. In various cancers, NF-κB activation is a sign of poor treatment response and worse patient survival. Activation of NF-κB occurs through multiplex upstream mechanisms. Administration of NF-κB inhibitors to the cancer cells via NPs that are capable of directly inhibiting NF-κB activation by the proteasomal degradation of NF-κB seems an important strategy for the treatment of breast cancers in which NF-κB is over-activated. These insights may provide novel therapeutic strategies to improve the outcome of treatment of breast cancer patients.

VYXEOS is the first nanoparticle drug carrier encapsulated into the liposome with a synergistic combination of free cytarabine and daunorubicin chemotherapeutic agents and approved by the FDA in 2017. Krauss et al. first clinically applied it in the treatment of acute myeloid leukemia. VYXEOS made a significant contribution to survival compared to other treatment regimens in which these drugs are applied freely^60^. It was previously known that these drugs destroy cancer cells with the DNA synthesis inhibition strategy. In our study, we showed that cancer cells go to apoptosis through a different strategy, targeting DNA synthesis with Dox and inhibition of the proteosome with Epo. For this reason, we think that lipid or polymeric nanoparticle formulations can be created using combination chemotherapy regimens commonly applied in the clinic or with new combinations of different drugs. Moreover, Patisiran/ONPATTRO (the first FDA approved siRNA therapeutic in 2018) is an siRNA-delivering lipid-based nanoparticle. Adams et al. developed that for the silencing of a specific gene responsible for the expression of transthyretin, which can cause hereditary transthyretin amyloidosis. Serum transthyretin levels decreased significantly in patients who were administered Patisiran / ONPATTRO intravenously compared to the placebo group^61^. On the other hand, mRNA-carrying lipid nanoparticle vaccines were developed to prevent COVID-19 infection and were produced very quickly thanks to nanobiotechnology in 2020. BioNTech / Pfizer and Moderna mRNA COVID-19 vaccines encode the viral spike glycoprotein of SARS-CoV-2 and promote immunity^62,63^. Although there is no polymeric nanoparticle used for clinical treatment at the moment, polymeric nanoparticles may be more likely to be used in the clinic in the future due to the fact that pharmaceutical and clinical formulations can be reproduced more rapidly and are more durable than lipid nanoparticles^64^.

## Conclusion

The idea of treating incurable diseases with nanoparticle-based drugs has recently attracted everyone's attention. In particular, drugs or bioactive molecules carrier systems developed with nanobiotechnology have been widely used in the treatment of many diseases such as cancers and viral infections. In addition, viruses and exosomes are nano-sized and can be internalized by cells and can cause many diseases. NPs can carry targeted molecules to treat or diagnose by mimicking the characteristics of a living organism. Therefore, new treatment methods can be improved in polymeric-based NPs containing cancer or virus specific peptides prepared with our technique. On the other hand, NPs can increase the efficiency of many drugs that are used extensively in the clinic, and drug resistance can be overcome with double and triple drugs combined applications encapsulated in NPs. Moreover, it can be used in personalized therapy applications for the treatment of complicated diseases including cancer. So, it can be an important opportunity to successfully encapsulate a small amount of active peptide into the NPs. This study could provide a promising treatment strategy to overcome multidrug resistance and toxicity to normal tissues that can be applied in further clinical studies to enhance apoptosis in breast cancer cells.

## Materials and methods

### Materials

Doxorubicin hydrochloride (Dox, MW: 579.98 g/mol) and Epoxomicin (Epo, MW: 554.7 g/mol) were purchased from Lc Labs (Woburn, Massachusetts, USA) and Cayman (Ann Arbor, Michigan, USA), respectively. PLGA [Poly (d,l-lactic-*co*-glycolide)] (MW: 30,000–60,000 g/mol), BSA, ethanol, acetone, acetonitrile, dichloromethane and dimethyl sulfoxide (DMSO) were purchased from Sigma-Aldrich (St. Louis, USA). Ultrapure water (Millipore Simplicity 185, Molsheim, France) was used to prepare nanoparticles.

### Preparation of Dox and/or Epo loaded nanoparticles

NPs were prepared according to a multiple emulsion solvent evaporation technique. Briefly, 20 mg of Dox were dissolved in 10 mL of methanol to achieve a final concentration of 2 mg/mL. 25 µg of Epo was dissolved in 250 µL DMSO. 100 mg of PLGA was dissolved in 3 mL ethyl acetate for 18 h. BSA (10 mg/mL) was prepared in PBS. Dox and Epo were separately encapsulated within the polymer mixture at the ratios of Dox: PLGA: BSA-(1:5:12.5) and Epo: PLGA: BSA-(1:25:62.5), respectively. For co-encapsulation of Dox and Epo, they were encapsulated as a combination within the polymer mixture at the ratio of Dox + Epo: PLGA: BSA-(5 + 1:25:62.5). These ratios were found by performing optimization studies to find the best encapsulation yield in terms of physico-chemical properties of the NPs. Dox and/or Epo were added dropwise to the polymer mixture during vortexing and sonicated (Bandelin Sonopuls, Berlin, Germany) at 50% strength for 30 s. The mixture was added to the aqueous phase containing 10 mg / mL BSA as a stabilizer and sonicated for 2 min at 65% strength. The solvent in the mixture was evaporated for 25–30 min using an evaporator (Buchi Rotavapor R3, Flawil, Switzerland). Removal of ethyl acetate solvent resulted in the formation of solid nanoparticles. Then, the mixture was transferred into a 50 mL falcon tube and centrifuged at 24,000×*g* for 60 min (+ 4 °C). The supernatants were collected and analyzed for the calculation of encapsulation efficiency while the precipitated pellets were stored to measure particle size and zeta potential of NPs. Before lyophilization, 5% glucose solution was added to disperse the NPs. The NPs were transferred to 50 mL tubes and lyophilized at 49 °C and 5.8 × 10^–2^ mbar for 2–3 days using a freeze-dryer (Toros TRS 2/2v, Teknosem, Istanbul, Turkey) (Supplementary Data).

### Particle size and zeta potential analysis

It is necessary to determine that the particle size and distribution of the prepared NPs are homogeneously in the range of 100–200 nm in order to be able to direct them with passive targeting. The average particle size and size distribution of NPs were measured and determined as mean diameter (nm) and polydispersity index, respectively by dynamic light scattering (DLS) measurements performed in triplicate at 25 °C at 90 °C angle based on quasi-elastic light scattering (QELS) technique using Malvern Nano ZSP (Malvern Instruments Corp., Worcestershire, U.K.). Measurements were made on the instrument with 2 µL NPs sample added into 1 mL distilled water. In addition, zeta potential values of the NPs ​​should be at the same surface charge for NPs not to agglomerate. Zeta potential values of the NPs were measured using the Malvern Nano ZSP at 25 °C at 120 °C angle. The NPs were diluted with ultra-pure water before measurement. Zeta potential (mV) was expressed as the average of ten measurements.

### Characterization of surface morphology

It is necessary to determine that the shape and surface morphology of the prepared NPs are homogeneously in the range of 100–200 nm to be able to direct them with passive targeting. Therefore, the shape and surface morphology of the NPs were examined by a scanning electron microscope (SEM, FEI, Quanta Feg 250, Hillsboro, USA) at 20.00 K × magnification. The NPs were prepared on stubs and coated with gold and palladium mixture. 10 mg of the NPs were analyzed under a low vacuum at a distance of 8 mm and using a voltage of 5 kV. Images were analyzed using the Image J software program.

### Molecular characterization

All molecules entering the NPs cause molecular changes by making chemical bonds with each other, and specific groups are either exposed or lost. So, the NPs were characterized using ATR-FTIR Spectroscopy (Bruker Tensor 27, Ettlingen, Germany) equipped with potassium bromide (KBr) beam diffuser and deuterated l-alanine doped triglycene sulphate (DLaTGS) detector to obtain spectra. Instrument control and data acquisition were performed using the OPUS program (Version 7.2 for Windows, Bruker GmbH). ATR-FTIR spectra of NPs were recorded in the range of 4000 to 600 cm^−1^ with a resolution of 2 cm^−1^ accumulating 16 scans per spectra. As a background, the air spectrum was examined under the same conditions before all measurements. 2 mg of sample was placed in device. Free NPs were used as the reference. The aim was to reveal if the drugs could be successfully loaded in NPs.

### Characterization of thermal properties

#### Characterization by differential scanning calorimeter

The molecular energy in their chemical bonds is directly proportional to all molecules entering the NPs. So, energy and phase changes of free, Dox loaded and Dox/Epo loaded NPs were determined using differential scanning calorimeter (Q100, TA Instruments Inc., New Castle, DE, USA). To perform the analysis, 2 mg of sample was placed in hermetically sealed aluminum pan and heated from 20 to 270 °C at a rate of 10 °C/min with a constant nitrogen flow rate of 20 mL/min to measure the weight loss followed by decomposition. An empty hermetically sealed aluminum container was used as a reference.

#### Characterization by thermal gravimetric analysis

The thermal properties of all molecules in the NPs were used to determine if drugs entered the NPs. Therefore, the thermal stabilities of NPs were determined by TGA (SII Nanotechnology‐SII6000 Exstar TG/DTA 6300, Tokyo, Japan). For this purpose, 8 mg of sample was placed in a platinum vessel and heated from 0 to 700 ºC at a rate of 10 ºC/min with a nitrogen flow rate of 20 mL/min. An empty aluminum pan was regarded as reference.

### Determination of encapsulation efficiency

#### Encapsulation efficiency of Dox

The encapsulation efficiency was determined based on mass-proportion of the amount of Dox retained in the NPs to the amount of Dox loaded in the NPs. In this method, 5 mg of Dox loaded NPs and Dox-Epo loaded NPs were dissolved in 1 mL of DMSO. The mixture was then diluted tenfold with ethyl acetate: methanol solution prepared at the ratio of 9:1 (v/v) and centrifuged at 20,000×*g* for 5 min. The encapsulation efficiency was determined using a HPLC (Shimadzu LC-20AD, Kyoto, Japan) equipped with RF-10AXL fluorescence detector. Separations were conducted for 20 µL of samples on Sepax C18-H (4.6 mm × 250 mm, 5 μm HPLC) column under the following conditions: Mobile Phase: 0.1% Formic acid (in water) and 0.1% Formic acid (in acetonitrile, organic) at a flow rate of 1 mL / min, starting with 5% organic solvent and increasing to 65% for 20 min. Finally, the amount was reduced to 5% organic solvent at 20.1 min and the operation was terminated at 30 min. The amount of Dox was calculated according to a linear calibration curve of Dox (1–200 μg/mL, Ex, 490 nm; Em, 592).

#### Encapsulation efficiency of Epo

The encapsulation efficiency was determined by mass-proportioning of the amount of Epo retained in the NPs to the amount of Epo loaded in the NPs. The encapsulation efficiency was determined by using a system of Agilent 6530 LC–MS-QTOF (Agilent Corp., Santa Clara, CA, USA). For sample preparation, 5 mg of Epo-NPs and Dox-Epo-NPs were dissolved in 1 mL of DMSO, followed by dilution tenfold with ethyl acetate: methanol solution prepared at the ratio of 9:1 (v/v). After shaken vigorously, it was centrifuged at 20,000×*g* for 5 min. Separations were conducted for 20 µL of samples on Athena C18 column (3 mm, 2.1 mm × 100 mm column) under the following LC–MS-QTOF conditions: Solvent A: 0.1% H_3_PO_4_ (in acetonitrile, organic), Solvent B: 0.1% H_3_PO_4_ (in water), Gradient: 0.4 mL at a flow rate of 1 mL / min. The mass spectra were recorded by scanning over a range of *m/z* 25–1000. Epo was detected at 19.8 min [observed *m/z* = 555.2 (M ^+^ H)^+^, 577.6 (M ^+^ Na)^+^]. The amount of Epo was calculated based on linear calibration curve plotted according to Epo solutions prepared at concentrations ranging from 1 to 1000 ng/mL.

Encapsulation efficiencies for Dox and / or Epo were calculated according to the following formula:

$${\%}\,\,\mathrm{E}\mathrm{n}\mathrm{c}\mathrm{a}\mathrm{p}\mathrm{s}\mathrm{u}\mathrm{l}\mathrm{a}\mathrm{t}\mathrm{i}\mathrm{o}\mathrm{n}\,\,\mathrm{e}\mathrm{f}\mathrm{f}\mathrm{i}\mathrm{c}\mathrm{i}\mathrm{e}\mathrm{n}\mathrm{c}\mathrm{y}=\left(\frac{\mathrm{A}\mathrm{m}\mathrm{o}\mathrm{u}\mathrm{n}\mathrm{t}\,\,\mathrm{o}\mathrm{f}\,\,\mathrm{e}\mathrm{n}\mathrm{c}\mathrm{a}\mathrm{p}\mathrm{s}\mathrm{u}\mathrm{l}\mathrm{a}\mathrm{t}\mathrm{e}\mathrm{d}\,\,\mathrm{d}\mathrm{r}\mathrm{u}\mathrm{g}\,\,\mathrm{r}\mathrm{e}\mathrm{t}\mathrm{a}\mathrm{i}\mathrm{n}\mathrm{e}\mathrm{d}\,\,\mathrm{i}\mathrm{n}\,\,\mathrm{N}\mathrm{P}\mathrm{s}}{\mathrm{T}\mathrm{o}\mathrm{t}\mathrm{a}\mathrm{l}\,\,\mathrm{a}\mathrm{m}\mathrm{o}\mathrm{u}\mathrm{n}\mathrm{t}\,\,\mathrm{o}\mathrm{f}\,\,\mathrm{d}\mathrm{r}\mathrm{u}\mathrm{g}\,\,\,\,\mathrm{l}\mathrm{o}\mathrm{a}\mathrm{d}\mathrm{e}\mathrm{d}\,\,\mathrm{i}\mathrm{n}\,\,\mathrm{N}\mathrm{P}\mathrm{s}}\right)\times 100$$

### In vitro release studies

Release experiments were performed to determine the suitability of NPs for release in vivo conditions at normal physiological pH (7.4) and at cell lysosomal pH (5.5). Therefore, in vitro release experiment was designed and acetate (pH 5.5) and phosphate (pH 7.4) buffers were used as the in vitro controlled release medium. The NPs containing 25 mg of any drug were placed in 50 mL of the buffer solution and stirred at 370 rpm in a magnetic stirrer at 37 °C. The amount of active substance released at specific time intervals was determined based on injection of 0.5 mL of solutions through a polyvinylidene difluoride (PVDF) syringe with a capacity of 0.1 µm. The removed solution was replaced with a fresh buffer solution. Quantitation of the amounts of Dox and Epo was performed using HPLC and LC–MS-QTOF systems, respectively. % Release was calculated according to the formula:

$$\mathrm{\%}\,\,\mathrm{R}\mathrm{e}\mathrm{l}\mathrm{e}\mathrm{a}\mathrm{s}\mathrm{e}=(1-\frac{\mathrm{E}\mathrm{n}\mathrm{c}\mathrm{a}\mathrm{p}\mathrm{s}\mathrm{u}\mathrm{l}\mathrm{a}\mathrm{t}\mathrm{i}\mathrm{o}\mathrm{n}\,\,\mathrm{e}\mathrm{f}\mathrm{f}\mathrm{i}\mathrm{c}\mathrm{i}\mathrm{e}\mathrm{n}\mathrm{c}\mathrm{y}\,\,\mathrm{d}\mathrm{u}\mathrm{r}\mathrm{i}\mathrm{n}\mathrm{g}\,\,\mathrm{s}\mathrm{t}\mathrm{o}\mathrm{r}\mathrm{a}\mathrm{g}\mathrm{e}}{\mathrm{I}\mathrm{n}\mathrm{i}\mathrm{t}\mathrm{i}\mathrm{a}\mathrm{l}\,\,\mathrm{e}\mathrm{n}\mathrm{c}\mathrm{a}\mathrm{p}\mathrm{s}\mathrm{u}\mathrm{l}\mathrm{a}\mathrm{t}\mathrm{i}\mathrm{o}\mathrm{n}\,\,\mathrm{e}\mathrm{f}\mathrm{f}\mathrm{i}\mathrm{c}\mathrm{i}\mathrm{e}\mathrm{n}\mathrm{c}\mathrm{y}})\times 100$$

### Cell line studies

In this study, cell line studies were performed using cell lines as in vitro models for NPs screening and toxicity studies. In this respect, the Human Umbilical Vein Endothelial Cells (HUVECs) and Michigan Cancer Foundation-7 (MCF-7) cells derived from human metastatic breast cancer were obtained from the American Type Culture Collection (ATCC) and used as the normal and breast cancer cell lines, respectively. The cells were grown in T-25 or T-75 flasks containing complete Dulbecco's Modified Eagle Medium (DMEM, Gibco, Paisley, UK) composed of 10% fetal bovine serum (FBS, Gibco, MD, USA) and 1% penicillin–streptomycin (Gibco, Massachusetts, USA) via incubation at 37 °C, under 5% CO_2_, and 95% relative humidity. All chemicals were of reagent grade and solvents were of HPLC grade.

Normal HUVECs and MCF-7 cells were divided into the following experimental groups to conduct cell line studies:

1. Control

2. Free Dox

3. Free Epo

4. Free Dox + Free Epo

5. DOXIL (commercial doxorubicin drug-containing liposome nanoparticle)

6. Free-NPs (Drug-free PLGA nanoparticle)

7. Epo-NPs (Epo encapsulated in PLGA nanoparticles)

8. Dox-NPs (Dox encapsulated in PLGA nanoparticles)

9. Dox + Epo-NPs (Dox + Epo encapsulated in PLGA nanoparticles).

#### Cell viability assay

In order to determine the cytotoxic doses of the lyophilized NPs, they were first dissolved in PBS and the main stock concentrations were adjusted to 10 mg / mL. Then, HUVECs and MCF7 cells were subjected to NPs and diluted with cell medium to a concentration of 10 mg / mL. The IC_50_ values of the free drugs, drug-free NPs and drug-loaded NPs were determined by XTT (2,3-bis-(2-methoxy-4-nitro-5-sulfophenyl)-2H-tetrazolium-5-carboxanilide) viability test that was used to determine the proliferation of cells and the effect of NPs on cell viability.

We applied the protocol in our previous study^65^. Briefly, in order to determine viability of the cells at 24 h, 7.5 × 10^3^ cells were placed in each well of a 96-well plate. Then, the cells were incubated overnight at 37 °C in a 5% CO_2_ humidified atmosphere. XTT test results were calculated according to the manufacturer's protocol, as following: after incubation, culture medium was removed and washed with 200 µL of pre-warmed PBS. Then, 100 µL of culture medium and 50 µL of XTT solution were added to each well-plate containing the samples and the mixture was incubated at 37 °C in a 5% CO_2_ humidified atmosphere. After incubation for 4 h, percentage of cell viability was calculated based on optical absorbance recorded at 460 nm with reference to 630 nm using a microplate reader (Multiskan GO Microplate Spectrophotometer, Thermo Fisher Scientific, Waltham, MA, USA). The values were expressed as percentage viability in proportion to the control group. IC_50_ values were calculated using dose effect table by plotting curve of each sample. IC_50_ values for HUVECs and MCF-7 cells were coded as H-IC_50_ and M-IC_50_, respectively. All experiments were performed using doses of M-IC_50_ obtained from cytotoxicity assays for MCF-7 cells. The aim was to determine the doses of administered NPs with the minimum toxic effect on normal cells but providing the maximum toxic effect on breast cancer cells.

#### Measurement of Intracellular Dox uptake

Increase in the amount of Dox is an important parameter indicating that it enters the cell with NPs containing Dox. The drug Dox is a fluorescent molecule, and it was used to measure relative cellular Dox levels. 25 × 10^4^ HUVECs and MCF-7 cells were placed in each well of 6-well plate and incubated with M-IC_50_ doses of all groups for 6 h, then washed and suspended in FBS-free DMEM. The cell suspensions were centrifuged at 500×*g* for 10 min; medium was removed, and cells were dissolved in 500 μl PBS. 100 μl of this cell mixture was added to each well of a black top reading plate for Dox measurement. Fluorescence was measured using a fluorescence microplate reader (Elx 800, Bio-TEK. Instruments Inc., Winooski, VT, USA) at Excitation 480 nm; Emission 592 nm. The amount of Dox entering the cells was calculated by subtraction of blank values for both cells to avoid light scattering from the cells. This measurement was performed at least 3 times for all Dox-containing groups.

#### Measurement of intracellular ROS

The amount of intracellular ROS is an important parameter showing that drugs enter the cell with NPs. The redox balance of cells is disrupted, and apoptosis accelerates with the increase in ROS level. Therefore, the average level of intracellular ROS in the HUVECs and MCF-7 cells was determined using cells loaded with the redox-sensitive dye DCFH-DA as an indicator of redox imbalance (Sigma, Schnelldorf, Germany). To measure the intracellular ROS level of the cells, 25 × 10^4^ HUVECs and MCF-7 cells were placed in each well of 6-well plate and incubated with M-IC_50_ doses of all groups for 18 h. The cells were washed twice in PBS, stained with 50 μM DCFH-DA in dark for 30 min and harvested at 37 °C. The cells were lysed with 1% Triton X-100, and fluorescence was measured using the fluorescence microplate reader at Excitation 485 nm; Emission 530 nm. A duplicate culture subjected to the same treatments was used to determine the total protein levels. The ROS levels were expressed as arbitrary unit/mg protein; as the percentage of control.

#### The 20S proteasome activity assay

The 20S proteasome activity measurement was used to determine whether Epo entered the cell with the NPs. The decrease in activity is an important indicator that shows Epo entering the cell. For this purpose, we isolated the cytosolic fractions of all cells. Cytosolic extracts (without protease inhibitors) were used to measure proteasome activity using 20S proteasome assay kit (BioVision, California, USA) applying the manufacturer's instructions. The assay is based on detection of the fluorophore 7-amino-4-methylcoumarin (AMC) after cleavage from the labeled substrate N-Succinyl-Leu–Leu–Val–Tyr-7-amino-4-methylcoumarin (Suc-LLVY-AMC). To measure the proteasome activity of the cells, 4 × 10^5^ HUVECs and MCF-7 cells were placed in each well of 6-well plate and incubated with M-IC_50_ doses of all groups for 24 h. At the end of the incubations, the medium was discarded, and the cells were washed with PBS. Cells were removed with trypsin, centrifuged and the supernatant was discarded. The substrate and buffer containing 50 mM 4-(2-Hydroxyethyl) piperazine-1-ethanesulfonic acid–potassium hydroxide (HEPES–KOH) at pH 7.5 were mixed to achieve a final volume of the collected pellet as 10 mg protein in 100 μl reaction volume. Levels of released AMC were measured using the fluorescence microplate reader at Excitation 380 nm; Emission 460 nm. Fluorescence values of the samples were measured at 37 °C for 1 h. Activity was calculated from the slope of the graph obtained from plotting of fluorescence values versus incubation time and the activities of the groups were compared. Background activity was determined using MG-132 at a final concentration of 10 mM. The relative activity was standardized by protein concentration and determined using Coomassie Protein Assay Reagent (Pierce, Rockford, IL, USA)^45^.

#### Cytosolic NF-κB-p65 assay

The cytosolic NF-κB-p65 measurement was used to determine whether Epo entered the cell with NPs. The increase of cytosolic NF-κB indicates that Epo is entering the cell via NPs and the cell will undergo apoptosis. For this purpose, we isolated the cytosolic fractions of all cells. The Enzyme linked immunosorbent assay (ELISA) was performed to accurately determine the presence of human NF-κB-p65 in cell lysates according to the protocol suggested by the manufacturer (Invitrogen, California, USA). Briefly, 3 × 10^6^ HUVECs and MCF-7 cells were placed in 75 cm^2^ flasks. M-IC_50_ was added to all groups except for the control at relevant doses and they were incubated for 24 h. The cells were collected by centrifugation at 600×*g* for 5 min at + 4 °C. The cell pellets were washed once with ice-cold PBS on ice. The cells were resuspended and lysed in Cytosol Extraction Buffer (10 mM Tris, 2 mM Na_3_VO_4_, 20 mM Na_4_P_2_O_7_, 100 mM NaCl, 1 mM NaF, 1 mM EGTA, 1 mM EDTA, 1% Triton X-100, 10% Glycerol, 0.1% SDS, 0.5% Deoxycholate, 1 mM PMSF, and protease inhibitor cocktail at pH 7.4.) on ice for 30 min. The homogenates were centrifuged at 10,000×*g* for 30 min at + 4 °C. The supernatants containing the cytosolic fraction and other subcellular constituents were collected. The NF-κB ELISA Assay Kit was used to obtain cytosolic NF-κB fraction. Absorbance values of known NF-κB-p65 standards were plotted over the values measured at 450 nm. The NF-κB protein levels in the samples were calculated based on their corresponding absorbance values using the standard curve.

#### Analysis of Annexin V using flow cytometry

The Annexin V-PE method was used to determine early apoptosis in the cell within the first 6 h after incubation, both of the free drugs and the NPs. For detection of apoptosis, the Annexin V-PE binding capacity of the treated cells was examined by flow cytometry using Annexin V-PE Detection Kit following the manufacturer’s protocol. Briefly, as outlined in our previous studies^66,67^, 5 × 10^5^ cells were placed in each well of a 6-well plate. M-IC_50_ was added to all groups except the control at relevant doses and they were incubated for 6 h as early apoptosis time. The cells were washed twice in PBS and then stained with Annexin V and 7-AAD. After stained with Annexin V and 7-AAD, the samples were analyzed by Muse flow cytometer (Luminex B.V. Hertogenbosch, Netherlands).

#### TUNEL analysis

TUNEL method was used to determine late apoptosis in the cell within the first 11 h after incubation, both of the free drugs and the NPs. We applied the protocol in our previous study to image apoptotic cells^67^. TUNEL Apoptosis Assay was used to detect apoptosis in individual cells using the TUNEL Detection Kit by following the manufacturer’s protocol (ScienCell, Carlsbad, CA, USA). Briefly, 1 × 10^4^ cells were placed in each well of a 96-well plate to adhere one day prior to incubation. M-IC_50_ were added to all groups except the control at relevant doses and incubated for 11 h as late apoptosis time. After the incubation medium was aspirated and the cells were washed twice in PBS, the cells were fixed with 4% paraformaldehyde for 10 min. Paraformaldehyde was removed and washed 3 times with PBS. Permeabilization was performed with 0.2% Triton X-100 prepared in PBS at room temperature for 15 min. The permeabilization solution was discarded and washed 2–3 times with PBS. The cells were incubated at room temperature for 10 min with equilibration buffer at 50 µL per well. TUNEL reaction mixture was prepared with 5 μl terminal deoxynucleotidyl transferase (TdT) solution and 45 μl Biotin-dUTP solution for each well to be studied. Each well was incubated with 1X TdT equilibrium buffer at 37ºC temperature for 60 min. Attention was paid to protection from light. The buffer solution was discarded and washed 3 times with PBS at two minute intervals. For the inactivation of endogenous peroxidases, 100 µL of 3% H_2_O_2_ solution per well prepared in PBS was incubated for 5 min at room temperature. The 3% H_2_O_2_ solution was discarded and washed 3 times with PBS at an interval of two minutes. Streptavidin-HRP solution prepared with PBS was added to each well and incubated at room temperature for 30 min. The solution was discarded and washed 3 times with PBS at two minute intervals. The stock 20X DAB substrate solution was diluted to 1X with PBS containing 0.3% H_2_O_2_. It was incubated in the dark and at room temperature until it turned brown-black. At the end of the incubation, the solution was discarded and washed 3 times with PBS with an interval of two minutes. The nuclei of apoptotic cells were observed as dark brown-black under an inverted phase contrast microscope (Leica DMIL, Germany). To obtain a quantitative standard for determination of apoptotic cell death, morphometric analysis was performed on the experimental groups.

### Statistical analysis

All experiments were performed in triplicate. Results were normalized to control group without scaffolding. Cell culture data are presented as the mean ± standard error (mean ± SE). Statistical analysis was performed using SPSS Data Access Pack for Windows version 23.0 (SPSS, Inc., Chicago, IL). p ≤ 0.05 was considered 95% confidence limits as a significant difference. Categorical and continuous variables were compared using the chi-square test, ANOVA, and Student’s t-test. The Mann–Whitney U-test and Kruskal–Wallis test were used to compare non-parametric variables. All experiments were performed in triplicate.

## Supplementary Information

Supplementary Figures.
