# Supplementary material for: A new combination strategy to enhance apoptosis in cancer cells by using nanoparticles as biocompatible drug delivery carriers
Source: Sci Rep. 2021 Jun 22;11:13027. doi: 10.1038/s41598-021-92447-x (PMC8219778; doi:10.1038/s41598-021-92447-x)
Supplement: Supplementary file 1 — Supplementary Figures. [file 41598_2021_92447_MOESM1_ESM.pdf]

A new combination strategy to enhance apoptosis in cancer cells by using nanoparticles as biocompatible drug delivery carriers

Kucuksayan, Ertan [1,5]; Bozkurt, Fatih [2,6]; Yilmaz, Mustafa Tahsin [2]; Sircan-Kucuksayan, Aslinur [3]; Hanikoglu, Aysegul [4]; and Ozben, Tomris [5]

[1]Alanya Alaaddin Keykubat University (ALKU), Faculty of Medicine, Department of Medical Biochemistry, Antalya, TURKEY

[2]Yildiz Technical University, Chemical and Metallurgical Engineering Faculty, Department of Food Engineering, Istanbul, TURKEY

[3]Alanya Alaaddin Keykubat University (ALKU), Faculty of Medicine, Department of Biophysics, Antalya, TURKEY

[4] Biruni University, Faculty of Pharmacy, Department of Biochemistry, Istanbul, TURKEY

[5]Akdeniz University, Faculty of Medicine, Department of Medical Biochemistry, Antalya, TURKEY

[6]Mus Alparslan University, Faculty of Engineering and Architecture, Department of Food Engineering, Muş , TURKEY

Corresponding author: Tomris Ozben; ozben@akdeniz.edu.tr

Akdeniz University, Faculty of Medicine, Department of Medical Biochemistry, Antalya, TURKEY

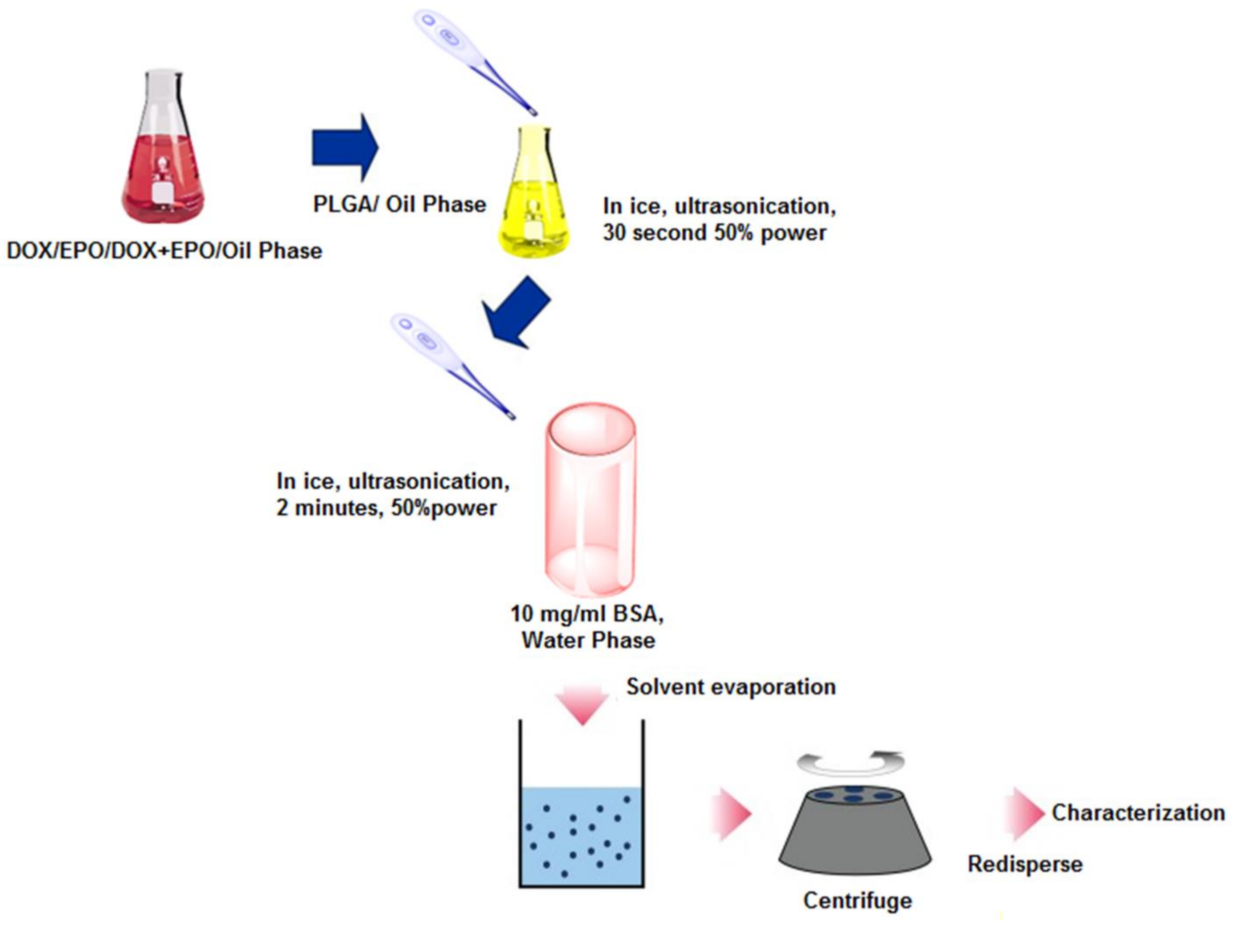

Supplementary Data 1. Summary of detailed preparation steps of nanoparticles.

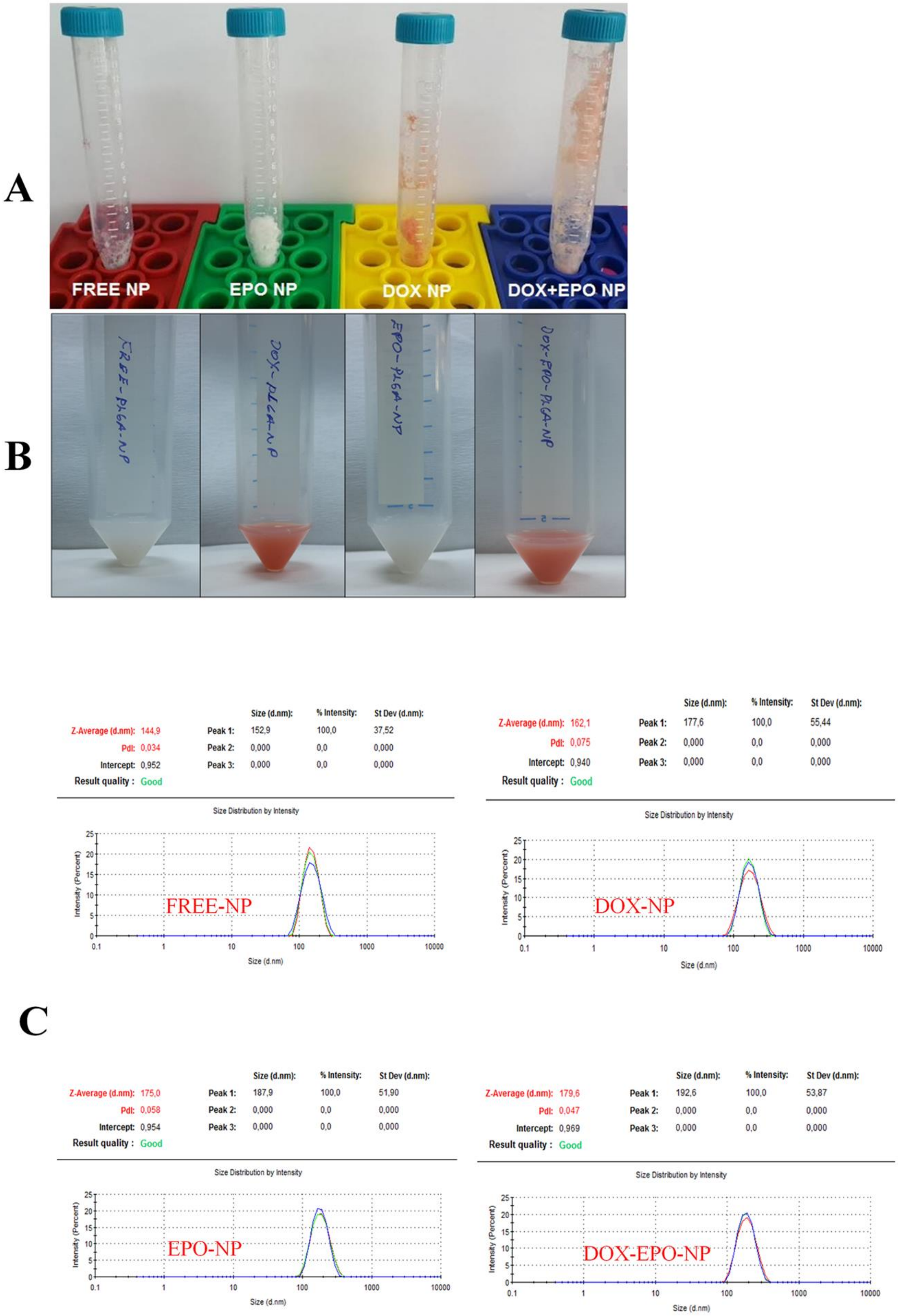

Supplementary Data 2. Images of the synthesized NPs. (A) after lyophilization, (B) after dispersed in PBS, (C) average particle size of the NPs.
